# Transcriptional Dynamics of the Embryonic Stem Cell Switch

**DOI:** 10.1371/journal.pcbi.0020123

**Published:** 2006-09-15

**Authors:** Vijay Chickarmane, Carl Troein, Ulrike A Nuber, Herbert M Sauro, Carsten Peterson

**Affiliations:** 1 Keck Graduate Institute, Claremont, California, United States of America; 2 Computational Biology and Biological Physics, Department of Theoretical Physics, Lund University, Lund, Sweden; 3 Lund Strategic Research Center for Stem Cell Biology and Cell Therapy, Lund University, Lund, Sweden; 4 Department of Oncology, Lund University Hospital, Lund, Sweden; The Salk Institute, United States of America

## Abstract

Recent ChIP experiments of human and mouse embryonic stem cells have elucidated the architecture of the transcriptional regulatory circuitry responsible for cell determination, which involves the transcription factors OCT4, SOX2, and NANOG. In addition to regulating each other through feedback loops, these genes also regulate downstream target genes involved in the maintenance and differentiation of embryonic stem cells. A search for the OCT4–SOX2–NANOG network motif in other species reveals that it is unique to mammals. With a kinetic modeling approach, we ascribe function to the observed OCT4–SOX2–NANOG network by making plausible assumptions about the interactions between the transcription factors at the gene promoter binding sites and RNA polymerase (RNAP), at each of the three genes as well as at the target genes. We identify a bistable switch in the network, which arises due to several positive feedback loops, and is switched on/off by input environmental signals. The switch stabilizes the expression levels of the three genes, and through their regulatory roles on the downstream target genes, leads to a binary decision: when OCT4, SOX2, and NANOG are expressed and the switch is on, the self-renewal genes are on and the differentiation genes are off. The opposite holds when the switch is off. The model is extremely robust to parameter changes. In addition to providing a self-consistent picture of the transcriptional circuit, the model generates several predictions. Increasing the binding strength of NANOG to OCT4 and SOX2, or increasing its basal transcriptional rate, leads to an irreversible bistable switch: the switch remains on even when the activating signal is removed. Hence, the stem cell can be manipulated to be self-renewing without the requirement of input signals. We also suggest tests that could discriminate between a variety of feedforward regulation architectures of the target genes by OCT4, SOX2, and NANOG.

## Synopsis

One key issue in developmental biology is how embryonic stem cells are regulated at the genetic level. Recent high throughput experiments have elucidated the architecture of the gene regulatory network responsible for embryonic stem cell fate decisions in human and mouse. In this work the authors develop a computational model to describe the mutual regulation of the genes involved in these networks and their subsequent effects on downstream target genes. They find that the core genetic network incorporates the functionality of a bistable switch, which arises due to positive feedback loops in the system. Also, this switch behaviour is very robust with respect to model parameters. The switch and architecture by which the genetic network regulates the downstream genes, is responsible for either maintaining the genes responsible for self-renewal on, and genes involved with differentiation off, or the opposite outcome, depending on whether the switch is on/off, respectively. The model also provides several predictions which can lead to further understanding of the network. The methods employed are fairly standard and transparent which facilitates further uncovering in future experimental investigations of genetic networks.

## Introduction

Embryonic stem (ES) cells possess the unique property of being able to retain their capacity for self-renewal and potential to form cells of all three embryonic germ layers (endoderm, mesoderm, ectoderm). Understanding the factors that determine the ability of ES cells to maintain their self-renewal and pluripotency, and the interplay of these factors, is of utmost relevance for both developmental biology and stem cell research. Over the last two years, a trio of transcription factors (TFs) have emerged—OCT4, SOX2, and NANOG—which play a key role in determining the fate of ES cells [1–4]. Many of these findings appear to hold both for human and murine systems, but some distinct differences exist with regard to target genes (TGs) of these transcriptional regulators and external factors. For example, LIF and BMP4 are critical external factors which maintain mouse, but not human, ES cells in vitro [5–7]. In contrast, BMP4 induces trophoblast differentiation of human ES cells [7], and this is accompanied by downregulation of OCT4. Factors involved in maintaining and propagating human ES cells are bFGF, Activin A, and the BMP antagonist Noggin [8–10]. The wnt pathway is important for both murine and human ES cell maintenance [11]. We consider networks that apply to both mouse and human. For reasons of simplicity, we use the symbols SOX2, OCT4, and NANOG for both species.

Recently, two genome-wide studies [3,4] were performed, in which these TFs were localized at promoter regions of human and mouse ES cells. Several transcriptional bindings were discovered: NANOG binds to the promoter regions of OCT4 and SOX2, as well as to its own. In addition, the OCT4–SOX2 heterodimer regulates NANOG, OCT4, and SOX2 individually. What emerges is a compact network with self-regulation and positive feedback. As pointed out in [3,4] and revealed by another large gene perturbation study with mouse ES cells [12], OCT4, SOX2, and NANOG also regulate a number of other genes. Many of these TGs are themselves TFs, some are responsible for maintaining ES cells by controlling self-renewal and pluripotency, and others perform key developmental functions that include differentiation into extra-embryonic, endodermal, mesodermal, and ectodermal cell types [3]. Such genes, which are crucial for development, are found to be repressed in ES cells. Henceforth, when we refer to *differentiation genes,* we imply a set of genes that are upregulated upon differentiation of ES cells and are negatively regulated by NANOG, OCT4, and SOX2. Similarly, by *stem cell genes* we mean those genes that are expressed in ES cells and are positively regulated by the three TFs. Thus, in this study we concentrate on TGs regulated by all three factors.

The three TFs OCT4, SOX2, and NANOG thus regulate genes with two distinct and opposing functions: self-renewal and differentiation. The TGs are regulated both by OCT4 and NANOG individually, and by the combined effects of OCT4–SOX2 and NANOG [3,4]. Furthermore, since OCT4–SOX2 activates NANOG, the TGs are at the receiving end of a feedforward loop [13]. We explore the consequences of this architectural feature after establishing the dynamics of the core of the network.

**Figure 1:**
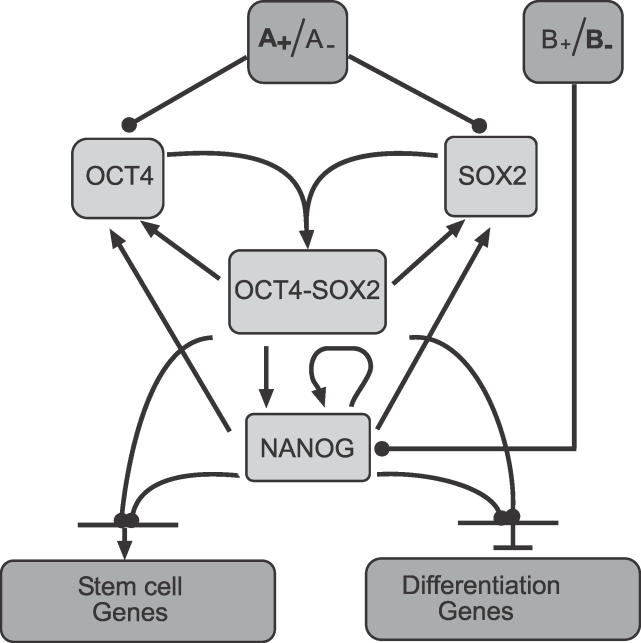
The Core Transcriptional Network of the ES Cell Genes and proteins are represented as single entities. Each outgoing arrow represents a protein (the outgoing merging arrows from OCT4 and SOX2 represent complex formation). Each ingoing arrow represents a protein with a role as a TF. Signals *A*_+_, e.g*.*, the wnt signaling protein, triggers the system to sustain self-renewal and pluripotency, whereas signals *B*_−_, e.g., p53, shuts it off, thereby leading to differentiation. It should be noted that there are also signals *A*_−_ that repress OCT4 and SOX2. These variants are not shown here since the effects will be similar to those of *B*_−_. OCT4, SOX2, and NANOG individually as well as jointly target stem cell and differentiation genes. We model the set of TGs that are jointly regulated by OCT4, SOX2, and NANOG. The nature of the regulation at the TGs is not specified, since we explore all the possibilities. However, the final effect of the regulation is indicated.

The core transcriptional network that emerges from [3,4,12] is depicted in Figure 1. We have investigated whether similar architectures exist in the gene regulatory networks of other organisms. Our study suggests that the architecture is unique to higher organisms, and it cannot be found outside the ES cell system from the available databases. In Figure 1 we include additional signals, denoted *A*_+_, *A*_−_, *B*_+_, and *B*_−_. Signals *A*_+_ and *A*_-_ positively and negatively regulate the expression of OCT4 and SOX2. The majority of Oct4 binding sites at TGs in ES cells are Sox–Oct composite elements [4,14] and here we consider OCT4 and SOX2 as common TGs of signals *A*_+_ and *A*_-_. Signals *B*_+_ and *B*_−_ positively and negatively regulate NANOG expression, respectively. We do not consider signals that act either positively or negatively on all three factors, since this situation is covered by the presence of *A*_+_ and *B*_+_ or *A*_−_ and *B*_−_. Several growth factors triggering certain signal transduction pathways required for keeping ES cells in an undifferentiated and multipotent state as well supporting their self-renewal have been described in the literature as mentioned above. These factors can be different for human and mouse ES cells. However, a signal pathway positively controlling both mouse and human ES cells is, for example, the wnt pathway (*A*_+_ or *B*_+_). It is currently not known which of the three TFs are directly regulated by wnt in ES cells. An example of a negative external signal (*A*_−_ or *B*_−_) is BMP4, which induces trophoblast differentiation in human ES cells [7]. In principle, signals *A*_+_, *A*_−_, *B*_+_, and *B*_−_ can also represent internal factors such as p53. p53 can be induced by DNA damage and negatively regulates NANOG in mouse ES cells [15]. It has been hypothesized that such a program gives the stem cell the opportunity to initiate differentiation, thereby reducing the impact of DNA damage [15].

We will discuss the dynamics of the transcriptional network as a function of the inputs *A*_+_, *A*_−_, *B*_+_, and *B*_−_. The operator sites on OCT4, SOX2, and NANOG at which these factors act are assumed to be far from the binding sites for OCT, SOX2, and NANOG, and hence no direct interactions between the factors and the trio of TFs occur.

We use the Shea–Ackers approach [16–20] to construct a stoichiometric model, which describes the dynamics of OCT4, SOX2, and NANOG. Using these tools we can describe the regulation of downstream TGs by OCT4, SOX2, and NANOG and explore the switch between self-renewal/pluripotency and differentiation—two very different outcomes.

The transcriptional network of Figure 1 contains positive feedback loops, which will turn out to give rise to bistable switch-like behavior, for a wide range of dynamical model parameters. Through the feedback loops, the expression of OCT4, SOX2, and NANOG can be jointly triggered or blocked by the environmental signals. We will argue that this switch-like behavior is highly useful in the context of stem cells, as it gives a clear separation of two very different cell fates: either maintain a stem cell state and differentiation is blocked or self-renewal and pluripotency of the ES cell is lost and differentiation is initiated. In Boolean terms, the stem cell genes should be on and the differentiation genes off, or the other way around, depending on whether the trio of TFs all are on or off. The model explains, qualitatively, several experimentally observed results concerning the behavior of OCT4, SOX2, and NANOG and their downstream TGs for maintaining an ES cell and for differentiation with respect to upstream signals. We use the model to generate several predictions. These predictions could be used to further validate the model or to pinpoint missing components.

## Results

Our system naturally divides into two parts: 1) The “stem cell box” with the tightly interacting OCT4, SOX2, and NANOG genes; and 2) their downstream targets—the stem cell and differentiation genes. After modeling each of these subsystems individually, the integrated model is described.

### The Stem Cell Box

#### Architecture.

In Figure 1 we consider the basic transcriptional circuit as described in [3]. We identify a central core, or stem cell box, consisting of OCT4, SOX2, and NANOG. OCT4 and SOX2 form a heterodimer, OCT4–SOX2, which positively regulates OCT4 and SOX2 [14] and NANOG [21]. NANOG, in turn, regulates both OCT4 and SOX2, and from perturbation studies this regulation appears to be of an activating nature [3,4]. NANOG also regulates itself and from [4] this autoregulation can be inferred to be positive. In summary, what emerges is a network with many positive feedback loops. In [14], the authors report two interaction motifs encountered in the mutual regulation of OCT4 and SOX2. One motif is autoregulation, where OCT4 and SOX2 regulate themselves. The other motif discussed is mutual activation, where OCT4 and SOX2 activate each other. This motif can sometimes establish switch-like behavior, where either both genes are on or both are off. In the case of the OCT4–SOX2 submodule in Figure 1, both these motifs stem from the formation of a heterodimer, OCT4–SOX2, which binds to the promoter regions of both SOX2 and OCT4. Furthermore, NANOG, which is activated by OCT4 and SOX2 through the action of the heterodimer, directly regulates OCT4 and SOX2 as an activator, thereby providing further positive feedback.

#### Phylogeny of architecture.

**Figure 2:**
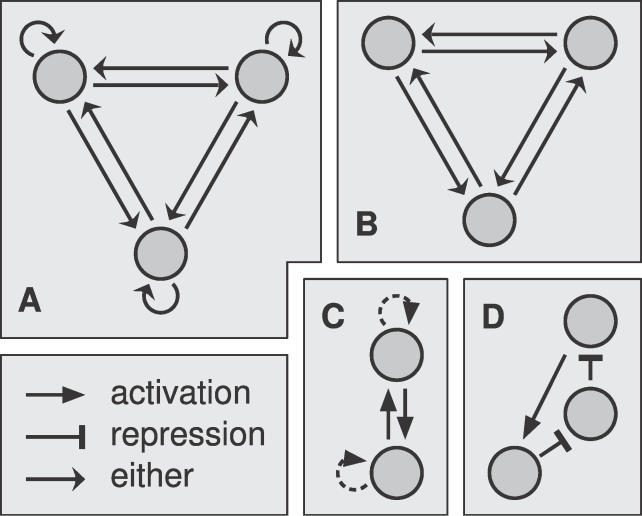
Putative Network Motifs Related to the System of OCT4, SOX2, and NANOG The motif in (A) captures the regulatory interactions of the system at the gene–gene level. Because (A) was not observed in any of the databases for lower organisms (see text), we searched for the same subgraph without autoregulations (B), followed by its two-gene counterpart with positive regulation (C). Finally, one of the activations was replaced with two repressions in series, yielding (D). A small number of instances of (C) and (D) were found in the databases (see text).

Prior to exploring the dynamics of the stem cell box, we examine whether its architecture occurs in the gene regulatory networks of other species. Such a search may lead to a better understanding of the network, if one could compare its functionality across different organisms. Our network motif searches are described in detail in Materials and Methods. The network in Figure 1 can be reduced to varying degrees to yield the stylized interaction networks shown in Figure 2. We searched for the occurrences of the networks in Figure 2 as subgraphs of the transcriptional networks of Escherichia coli [22], Saccharomyces cerevisiae [23], and Drosophila melanogaster [24]. In the available network data, little was found that looked anything like the stem cell box. The closest match was given by three *Drosophila* genes (Krüppel, Knirps, and Giant) whose interactions match those of Figure 2B. However, far from forming a compact module with positive feedbacks, these three fly genes form part of the highly connected gap gene network, responsible for setting up spatial patterns during early embryonic development [25,26]. This is achieved through a system of mutual activations and repressions, such that five of the six pairwise interactions between Krüppel, Knirps, and Giant are repressions. This leads to dynamics very different from those of the ES cell box.

A search for switch-like modules of only two genes produced the mutually activating genes CAT5 and CAT8 in yeast, and achaete and scute in *Drosophila*. In both cases, one of the genes is also activating itself, thereby strengthening the bistable behavior. We conclude that with available data on transcriptional networks from other species, one cannot find evidence for motifs resembling the central core of the ES cell box of Figure 1. Some phylogenetic investigations on the component level, i.e., on genes, revealed only weak homologies [3]. For example, for NANOG there is only a 50% homology with its chicken counterpart.

#### Dynamical model.

From the nature of the connections as described earlier in the Architecture section, we know that the heterodimer OCT4–SOX2 on its own serves as an activator for all three genes (OCT4, SOX2, and NANOG), and we surmise that it also works as an activator when in complex with NANOG. The model (see Materials and Methods) is based on cooperative binding of the heterodimer and NANOG to promoters, as is realized if the heterodimer first binds to a promoter and then recruits NANOG.

As explained in the Introduction, we also assume that the signals *A*_+_, *A*_−_, *B*_+_, and *B*_−_ act additively on OCT4, SOX2, and NANOG complexes, i.e., the signals do not directly interact with the three TFs in any way.

Furthermore, signal *A*_+_ has the effect of increasing the concentrations of OCT4, SOX2, and subsequently NANOG, through positive feedback loops, whereas the signal *A*_−_ has the opposite effect. Similarly signals *B*_+_ and *B*_−_ have opposite effects when they activate/suppress NANOG, respectively. In our calculations, we have sought to focus on *A*_+_ and *B*_−_, the former activating OCT4 and SOX2, and the latter suppressing NANOG. Since our model assumes a recruitment mechanism wherein an OCT4–SOX2 heterodimer first binds to the DNA and then recruits NANOG, it turns out that the option *B*_+_ is not very effective in increasing levels of OCT4, SOX2, and NANOG, if initially these start out at low values. However *B*_−_ is more effective in decreasing the levels of OCT4 and SOX2 through the positive feedback loop through NANOG. Hence we use *A*_+_ to activate the network, and *B*_−_ to deactivate it.

With the assumptions above, a set of differential equations emerge, which describe the behavior of OCT4, SOX2, NANOG, and OCT4–SOX2, with concentration levels [*O*], [*S*], [*N*], and [*OS*], respectively:

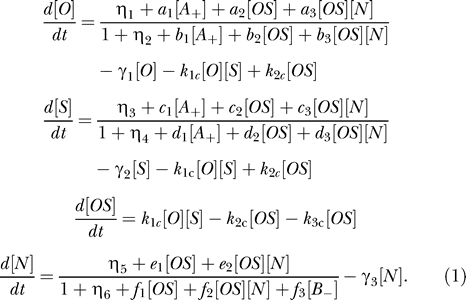



Here *a_i_, b_i_, c_i_, d_i_,* and *f_i_* are constants related to the free energies of binding of TFs to the operator regions of OCT4, SOX2, and NANOG. These implicitly take into account interactions between the TFs and/or RNA polymerase (RNAP). The basal transcription rates, which are thought to be low, are denoted by *η_i_,* while *γ_i_* and *k_ic_* denote decay rates and kinetic constants, respectively. The derivation of Equation 1 and the parameter values are found in Materials and Methods (Tables 1 and 2) and in Protocol S1. Macroscopic rate equations are appropriate here since the number of transcripts, known from SAGE and MPSS data [27,28], appears to be sufficiently high that stochastic effects can be neglected.

**Table 1:**
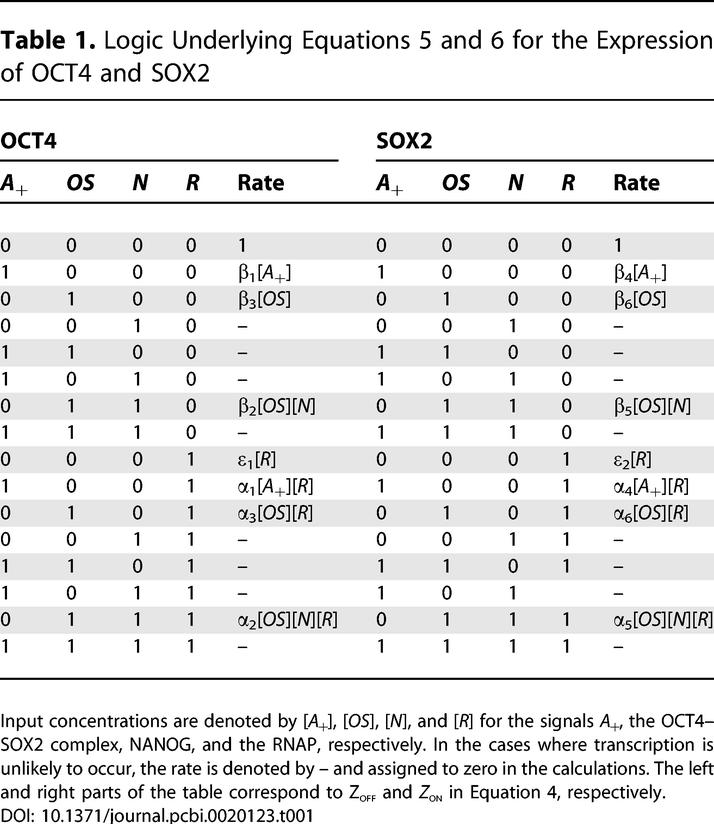
Logic Underlying Equations 5 and 6 for the Expression of OCT4 and SOX2

**Table 2:**
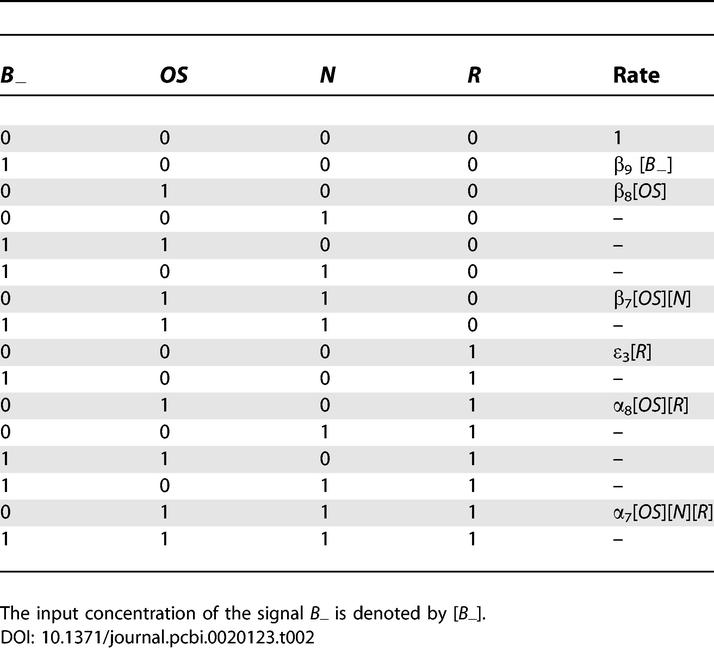
Logic Underlying Equation 7 for the Expression of NANOG with the Same Notation as in Table 1

**Figure 3:**
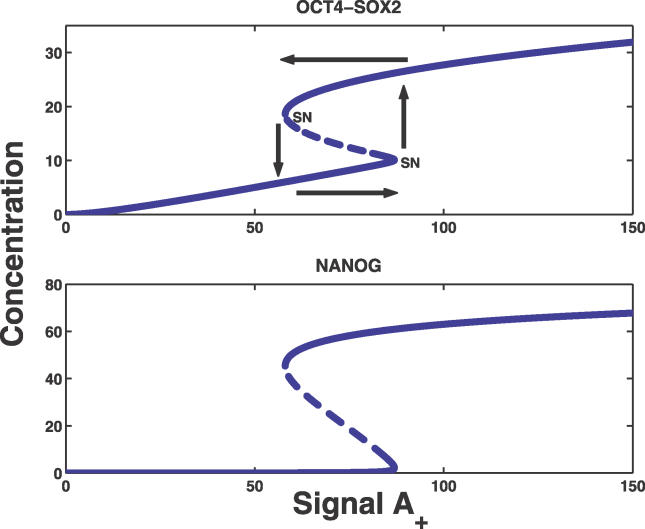
Steady State Behavior of the OCT4–SOX2 and NANOG Concentration Levels as Functions of the Signal *A*_+_ There are two turning points (saddle-node bifurcations are marked as SN, and the dotted line connecting the SNs indicate unstable states) leading to a hysteretic curve. The arrows indicate how to interpret the hysteresis curve. As *A*_+_ is increased beyond *A ≈* 87 (arbitrary units), the system switches on, and thereafter as the stimulus is decreased below *A ≈* 58, the switch turns off. The on/off states refer to high/low levels of OCT4, SOX2, and NANOG.

In Figure 3, we show the steady state concentrations of OCT4–SOX2 and NANOG, from Equation 1, as functions of *A*_+_ when *B*_−_ is kept at a low level. The corresponding curves for OCT4 and SOX2 (unpublished data) look qualitatively the same. The curves in Figure 3 demonstrate the system's bistable switch-like behavior with respect to the input signal *A*_+_. As *A*_+_ is increased, the levels of OCT4 and SOX2 start to grow, and this leads to the formation of more OCT4–SOX2, with the consequence that OCT4 and SOX2 are further activated. This positive feedback loop is further enhanced by NANOG, which is itself activated by OCT4–SOX2 and hence provides further positive feedback to OCT4, SOX2. At some threshold of the input signal *A*_+_, the positive feedback loops abruptly trigger all three genes to be turned on. Decreasing the signal will turn off the three genes again, but because of hysteresis this will happen at a lower threshold of *A*_+_. The positive feedback loop through NANOG determines these thresholds. The NANOG feedback to OCT4 and SOX2 is also amplified by the positive autoregulation of NANOG. As we will explain in the next section, this autoregulation plays an additional, important role in modulating the responses of the TGs.

**Figure 4:**
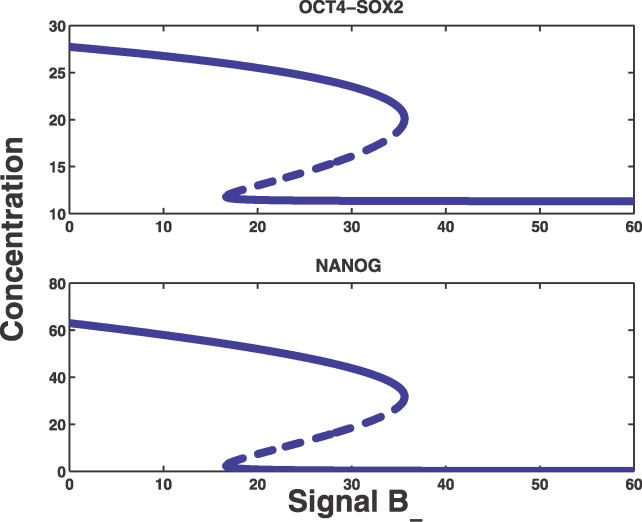
Steady State Behavior of OCT4–SOX2 and NANOG Concentration Levels as a Function of the Signal *B*_−_ The system is a bistable switch with respect to the *B*_−_ concentration. As the threshold is crossed (*B ≈* 36, arbitrary units), NANOG turns off, and OCT4 and SOX2 are reduced to lower levels. Since the signal is still present (*A*_+_ = 100), the OCT4 and SOX2 levels do not decline to very low levels.

Activating the signal *B*_−_ in Figure 1 will lower the level of NANOG, and then the feedback from NANOG shuts down OCT4 and SOX2. By this mechanism *B*_−_ can turn off the switch. The steady state curves in Figure 4 were obtained from initial conditions with the signal *A*_+_ present and at a level where the switch is on. We then compute the steady state values of OCT4–SOX2 and NANOG as functions of *B*_−_. As *B*_−_ is increased, NANOG is increasingly repressed, reducing its ability to positively regulate OCT4 and SOX2, and ultimately the switch is turned off. Due to the presence of the input signal *A*_+_, there is still some residual expression of OCT4 and SOX2, but at a much lower level than when the switch is on. The two states, i.e., OCT4, SOX2, and NANOG being on/off, allow TG regulation such that the expressions of stem cell and differentiation genes are mutually exclusive, in particular if the regulation makes use of all of OCT4, SOX2, and NANOG.

The concept of bistability in a genetic regulatory network has long been hypothesized to have a role in differentiation [29]. It seems likely that for cell-fate decision systems, especially in stem cells where distinct possibilities are faced by the cell, an external signal concentration can switch a system to its final state [30]. Bistability naturally leads to hysteresis, the retention of memory, whereby the cell can maintain its state even on removal of the external signal. Recent work [31,32] has experimentally demonstrated hysteresis in mammalian gene networks. In signaling and genetic networks, bistability arises from the nonlinearity and positive feedback loops of the protein–protein and protein–gene interactions [33–36]. Switch-like behavior is seen in most systems that have autoregulation, positive feedback, and mutually antagonistic interactions, as has been demonstrated in several synthetic circuits [37–39]. With regard to stem cells, several examples where autoregulatory TFs are involved in cell fate are discussed in [30]. In theoretical models that describe regulation of stem cells, autoregulatory and mutually antagonistic interactions have been argued to lead to switch-like behavior [31,40–42].

We have examined the robustness of switch-like behavior of the stem cell box to changes in parameter values (see Protocol S1 for details). The system exhibits bistability for a wide range of parameter values, such as TF binding efficiencies, degradation rates, and basal transcription rates. In particular, due to the positive feedback loops between OCT4, SOX2, and NANOG, the model predicts that increasing the binding efficiency of NANOG to OCT4 and SOX2 leads to stronger positive feedback, and then less activation from *A*_+_ is required to turn the switch on. For even higher values of the binding efficiency, the lower threshold in *A*_+_ for turning the switch off drops to zero, creating an irreversible switch (see Protocol S1).

In conclusion, the stem cell box has the interesting dynamics of being turned on by a signal, *A*_+_, exemplified by wnt in mouse and human ES cells, and turned off by an opposing factor, *B*_−_, which in mouse ES cells could be p53.

### Regulation of TGs

As mentioned before, the TGs of the stem cell box considered here are either regulated individually by OCT4–SOX2 and NANOG or by their joint actions [3,4]. In what follows we confine ourselves to the latter alternative. Experimentally it is known that when the three TFs are expressed, the stem cell TGs are on and the differentiation TGs are off, and vice versa. In what follows we will focus on TGs that are regulated by the joint actions of OCT4, SOX2, and NANOG as shown in Figure 1. From a dynamical standpoint, these TGs are more interesting than those regulated by only one of the three TFs.

**Figure 5:**
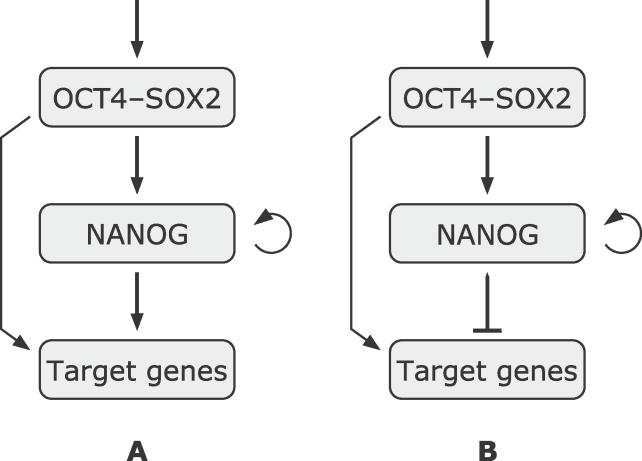
Coherent and Incoherent Feedforward Network Motifs (A) Coherent. (B) Incoherent.

The OCT4–SOX2 heterodimer regulates NANOG, and together OCT4–SOX2 and NANOG then regulate the TGs. This is a realization of the feedforward loop motif [22] (see Figure 5). Feedforward loops can lead to two types of dynamics depending upon the interaction between the two signals that control the TG. In the coherent case (Figure 5A), the feedforward loop can act as a low pass filter, only responding to persistent signals [13,43,44]. However, in the incoherent case (Figure 5B), the TG activity can react strongly to transients, since the suppressing signal is delayed by passing through NANOG from OCT4–SOX2. This has been suggested to be a general mechanism for improving response times in transcriptional networks [45]. Recently, it has also been argued that such incoherent feedforward loops could be very important in establishing spatial stripes and pulsed temporal expression profiles of TFs involved in developmental processes [46].

Whether the feedforward mechanisms governing the stem cell and differentiation genes are coherent or incoherent is not known. Here we explore the steady state properties of two different alternatives: I) stem cell genes: OCT4–SOX2 activator, NANOG a weak repressor; differentiation genes: OCT4–SOX2 activator, NANOG repressor; II) stem cell genes: OCT4–SOX2 activator, NANOG activator; differentiation genes: OCT4–SOX2 repressor, NANOG repressor.

The first case, which has the same architecture for regulating both types of TGs, is explored in some detail here. The second case will be commented upon in the next section.

To focus on the dynamics of the feedforward regulation of TGs, we will here assume a fixed OCT4–SOX2 concentration, i.e., exclude the effects of the feedback of NANOG to OCT4 and SOX2. In the next section we integrate the stem cell box of the previous section with the feedforward regulation.

We assume the following regulatory mechanism at the TGs: OCT4–SOX2 binds to the TGs as an activator and recruits NANOG. Together the OCT4–SOX2, NANOG complex then acts as a repressor for the TGs. For low OCT4–SOX2 concentrations, the TGs are activated. As the level is increased, the NANOG concentration grows, thereby enabling OCT4–SOX2 and NANOG to repress the TGs. The steady state response of this circuit is therefore maximal at an intermediate OCT4–SOX2 concentration, giving rise to a behavior of an “amplitude filter” [47,48]. The circuit functions as a “concentration detector,” providing maximal output for inputs within a certain range [46,49].

With constant input concentration [*OS*] to the system (Figure 5B), we obtain for the TG transcription rates:

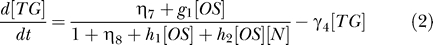



**Figure 6:**
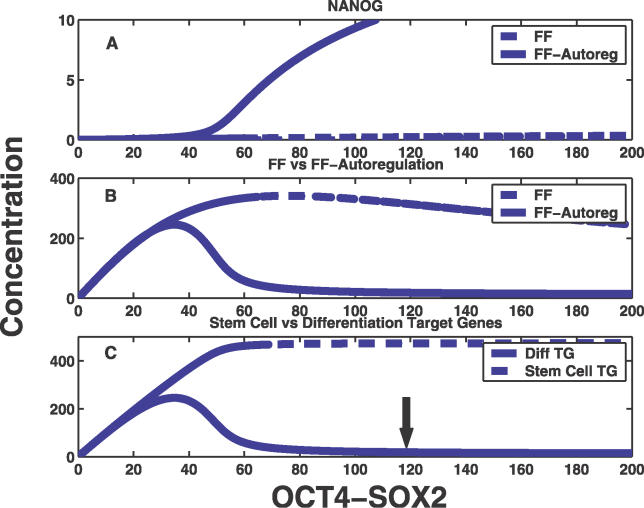
Steady State Concentrations of NANOG and the TGs as Functions of the Input OCT4–SOX2 Concentration [*OS*] for the Feedforward Motif with Autoregulation (FF-Autoreg) as well as the Feedforward Motif with No Autoregulation (FF) (A) NANOG. (B) TGs. (C) Concentrations of the stem cell and differentiation TGs as a function of [*OS*] are shown. The arrow in (C) indicates that if such a value of [*OS*] is reached, then the differentiation TGs could be shut off, whereas the stem cell TGs would be on. Thus the same incoherent architecture for both sets of genes achieves the desired result.

Equation 2 is derived in Materials and Methods, and the parameter values are found in Protocol S1. In Figure 6 we plot state values of NANOG concentration and the TG expression as functions of the OCT4–SOX2 concentration ([*OS*]). We also compare with these curves, the steady state values computed for a simple feedforward, i.e., NANOG does not have any autoregulation. In Figure 6A, as [*OS*] increases, NANOG is activated, and since it is autoregulatory, at some threshold of [*OS*], NANOG is fully turned on. NANOG then binds cooperatively with OCT4–SOX2 at the TGs, causing repression of those genes. This can be seen in Figure 6B, where we plot differentiation gene expression. We observe a phase of linear growth with [*OS*], when the TGs are activated and the level of NANOG is low, followed by a rapid decline when NANOG becomes highly activated. As expected, this system behaves like an amplitude filter, whereby the response of the differentiation TGs peaks at a certain input of [*OS*]. It is interesting to compare this response with the case where NANOG lacks autoregulation, i.e., a simple feedforward network (see Protocol S1 for parameter values). Figure 6A for this case shows that NANOG levels are fairly low, and in Figure 6B the differentiation TG expression, although showing a peaked behavior, is significantly wider compared with the case where NANOG is autoregulatory. Hence, with a simple feedforward network, it is not possible to adequately suppress the differentiation TGs. In Figure 6C, we compare the steady state behavior of the stem cell gene expression with that of the differentiation genes, when the binding of NANOG to OCT4–SOX2 is weaker at the stem cell genes than at the differentiation genes. As can be seen, the expression of the differentiation TGs vanishes at the same time as expression of the stem cell TGs saturates, even though the regulatory mechanisms are qualitatively the same. The role of NANOG autoregulation in the incoherent feedforward loop is to provide a sharper cutoff, leading to a narrower amplitude bandwidth, for the differentiation gene expression as a function of [*OS*]. These characteristics, which are achieved by one and the same architecture, are summarized as follows.

#### Stem cell genes.

NANOG is recruited weakly by OCT4–SOX2, causing only minor repression of the stem cell gene, so that it is expressed at a significant level. The binding strengths and the concentration of [*OS*] determine the final expression level as is described by the amplitude filter curve (Figure 6C).

#### Differentiation genes.

NANOG binds with higher affinity to OCT4–SOX2, so that the OCT4–SOX2, and NANOG complex are very effective at repressing the differentiation genes. When the [*OS*] concentration level is much higher than the optimum level (i.e., at the [*OS*]-value at which the TG expression peaks), the TGs are completely repressed.

### Integrating the Parts

**Figure 7:**
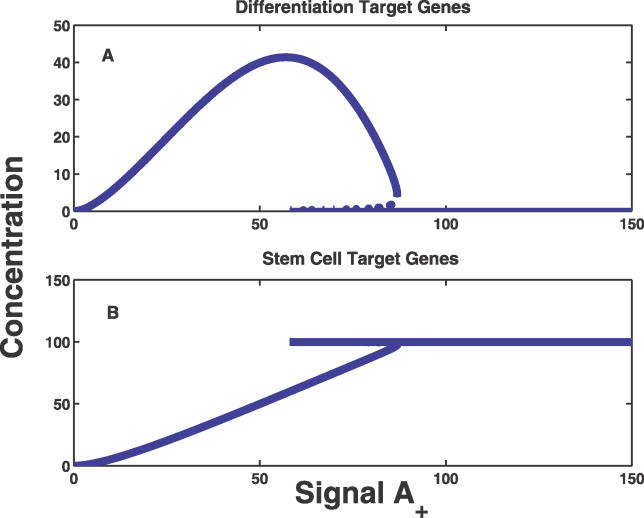
Steady State Values of the TG Expression as a Function of the Input Concentration *A*_+_ for the Differentiation and Stem Cell Genes Using for the Integrated Model with the Incoherent Feedforward Architecture Differentiation TG Expression and Stem Cell TG Expression (A) Differentiation TG expression. (B) Stem cell TG expression. Once the stem cell box is on, the [*OS*] levels are fixed by the switch: for example, if it is larger than *≈*100, the stem cell genes are expressed and the differentiation genes are shut off.

We next integrate the stem cell box with the feedforward regulation of the TGs by closing the loop, i.e., NANOG feeds back to the OCT4 and SOX2 genes. The OCT4–SOX2 input to the feedforward motif is now a variable determined by its interaction with the signals *A*_+_ or signal *B*_−_ and NANOG. With the same incoherent feedforward architecture for both sets of TGs, as discussed earlier, Figure 7 shows the expression levels as functions of *A*_+_. Figure 7A shows how the differentiation gene expression grows almost linearly with *A*_+_, but drops off above a certain level of the input signal *A*_+_. At this threshold, the stem cell box is turned on, and OCT4, SOX2, and NANOG rise to high levels, and cooperatively bind to the TGs to repress them. We note the sudden sharp drop of the amplitude filter curve, due to the bistable switch behavior, which ensures almost complete repression of differentiation TGs. Figure 7B shows the steady state values of the stem cell genes, which differ from the differentiation genes of Figure 7A only by the lower binding efficiency of NANOG (see Protocol S1 for parameter values). At a certain threshold of *A*_+_, the stem cell box is turned on and the concentrations of OCT4–SOX2 and NANOG saturate at high levels. Since NANOG only effects weak repression of the self-renewal genes, the activation by OCT4–SOX2 dominates. Thus, the self-renewal genes are highly expressed at a certain threshold of *A*_+_, as determined by the amplitude filter curve (Figure 6).

**Figure 8:**
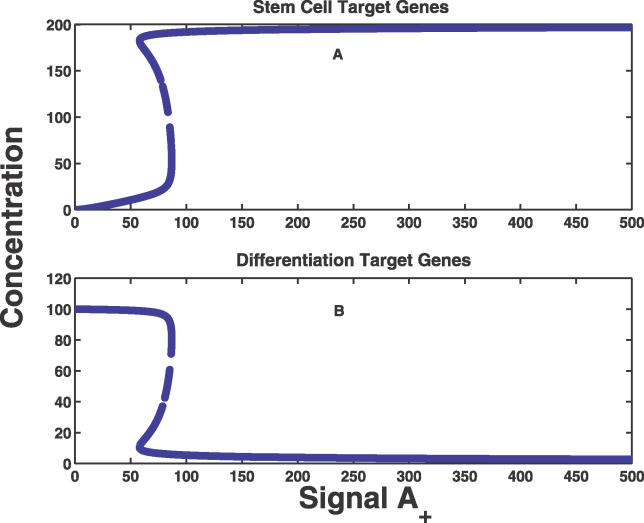
Steady State Values of the TG Expression as a Function of the Input Concentration *A*_+_ for Stem Cell and Differentiation Genes, Respectively, for Case II (A) Stem cell genes. (B) Differentiation genes.

Next we consider case II for the regulation of the TGs. Here the stem cell genes are regulated with both OCT4–SOX2 and NANOG being activators, and differentiation genes are regulated with both OCT4–SOX2 and NANOG being repressors. In Figure 8A we show the stem cell TG expression, which as expected shows a sudden rise to an almost constant value, as a function of the input signal. Figure 8B shows the differentiation TG expression being shut off at a certain threshold of the input signal *A*_+_. Equations 1 and 2 were used to generate the curves in Figure 8 with a modification in Equation 2 consistent with the type of regulation considered (see Table 3B and Protocol S1).

**Table 3:**
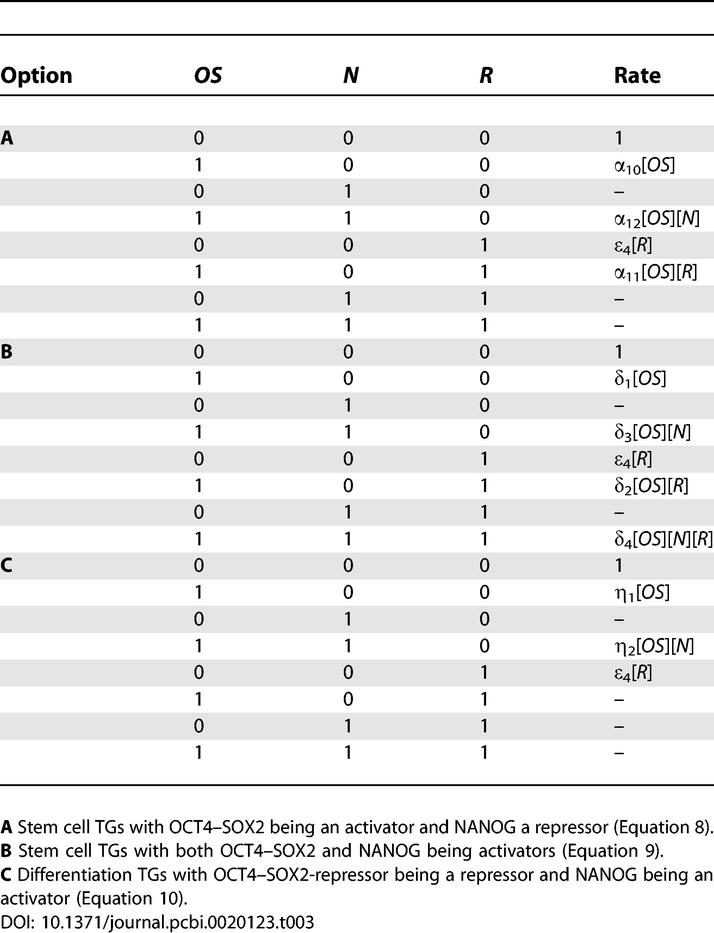
Logic for the TG with the Same Notation as in Table 1

Both cases I and II comply with the fact that the stem cell genes are expressed and differentiation genes are repressed when the switch is on, and vice versa. We propose possible tests to distinguish between the cases in the Discussion. In case I, both sets of TGs are controlled by the same architecture, and one might speculate that this is more likely from an evolutionary standpoint.

#### Effects of weak feedback from NANOG to OCT4 and SOX2.

**Figure 9:**
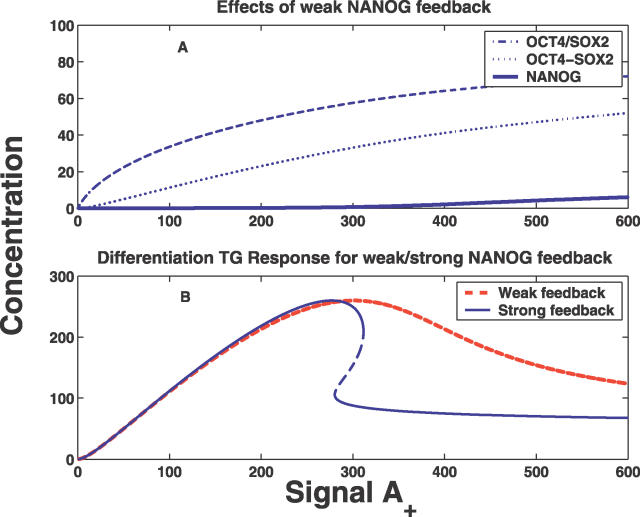
Steady State Values of TG Expression as a Function of the Input Concentration *A*_+_, for the Differentiation and Sel-Renewal Genes for the Case when NANOG Binds Weakly/Strongly to the OCT4 and SOX2 Genes (A) The effects of weak feedback of NANOG to the OCT4 and SOX2 genes. The inefficient binding of NANOG to these genes leads to a loss of the switch-like behavior. (B) The consequence of weak NANOG binding on the TG expression. With weak feedback, the expression level does not fall off sharply as compared with the case with strong feedback, thereby demonstrating that strong feedback is essential to prevent the differentiation TGs from being expressed.

We now turn to the effects of reducing the feedback from NANOG to OCT4 and SOX2 on the regulation of the TGs. This is illustrated using the incoherent feedforward architecture (case I). If the binding strengths of NANOG to OCT4 and SOX2 are decreased, the positive feedback is reduced and the bistable behavior is lost, which affects the expression levels of the TGs. Figure 9A shows OCT4, OCT4–SOX2, and NANOG for weak bindings of NANOG to OCT4 and SOX2 as a function of *A*_+_. Each of these rises steadily but with no switch-like behavior. In Figure 9B we show the response of the differentiation TGs (dashed line) using the same parameters. Note the relatively slow fall of the curve, which indicates that the differentiation TGs cannot be in a repressed state even for a large signal *A*_+_. In comparison, Figure 9B shows the response of the TGs genes (solid line) for the case where the feedback of NANOG to OCT4–SOX2 is strong. The switch-like behavior is now recovered, which ensures a more rapid falloff of the TGs. Weak feedback from NANOG to OCT4 and SOX2 can be counterbalanced by increasing the autoregulatory feedback of NANOG, since increased NANOG levels are then available for binding to OCT4 and SOX2 thereby restoring the positive feedback loop. We have confirmed this behaviour with simulations (unpublished data). Hence, feedback of NANOG to OCT4 and SOX2 is complementary to NANOG autoregulation, and in a sense they are both “fallback” options to recovering the switch-like behavior.

## Discussion

The ES cell transcriptional network architecture appears to be unique. Our data analysis has shown that the transcriptional subnetwork between OCT4, SOX2, and NANOG has no counterparts in available information from non-mammalian genomes. Hence, one cannot rely on phylogenetics to confirm and deduce the function of the architecture. Instead, dynamical modeling of the system is called for [33,47]. To this end, a continuous differential equation model based upon the Shea–Ackers formalism [18] was employed, and using this we have developed a model for the core transcriptional dynamics of ES cells involving the OCT4, SOX2, and NANOG genes, their self-regulation, as well as their regulation of stem cell and differentiation TGs.

We have identified a bistable switch in the network consisting of the OCT4, SOX2, and NANOG genes, which arises due to positive feedback loops: OCT4 and SOX2 regulate each other through the formation of a heterodimer. This heterodimer regulates NANOG, which feeds back to OCT4 and SOX2. NANOG also binds to its own promoter region as an activator, and this autoregulation acts to further strengthen the positive feedback loop. The two states of the stem cell box switch, where the trio of TFs are all on or off, correspond to stem cell and differentiation, respectively. The switch was found to be robust with respect to model parameters (see Protocol S1), a property arising from the architecture. The stem cell box can be switched on by a signal *A*_+_, where examples may include wnt for mouse and human ES cells and LIF for mouse ES cells. Suppression of NANOG by the signal *B*_−_, exemplified by p53 in mouse, turns the switch off. We speculate that it is perhaps more practical to use *B*_−_ to turn the switch off, than to remove the signal *A*_+_, since external growth factors positively regulating the three TFs might still be present in the environment of an ES cell when it starts to differentiate. One way this can be achieved is that DNA damage in mouse induces p53, which can downregulate NANOG, and ultimately induce differentiation. Recent experiments [4] show that the transcriptional stem cell network for mouse is similar to that for human ES cells [3]. It is interesting to speculate whether p53 has the same effect in human ES cells, i*.*e*.*, repressing NANOG and shutting off the stem cell box, thereby leading to differentiation. Since our model applies to both mouse and human ES cells, several of the generic predictions that the model offers can be tested in either organism.

Next we considered the regulation of TGs by OCT4, SOX2, and NANOG, pointing out that if the TGs are regulated individually by OCT4, SOX2, and NANOG, then the stem cell box dynamics takes care of keeping the stem cell genes on and the differentiation genes off. We therefore focused on those TGs that are jointly regulated by the OCT4–SOX2 heterodimer and NANOG.

Two options for the regulation of TGs were modeled, and we found that both were consistent with the requirement that stem cell genes are on and differentiation genes are off when the stem cell box is on. Below we suggest possible tests to discriminate between the two options. We also argue that an incoherent feedforward loop with autoregulation gives rise to an “amplitude filter” effect, which provides the required repressive behavior of differentiation TGs when the switch is on, and a stable expression level for the stem cell TGs if the binding of NANOG to the TG is weak. Both sets of genes can thus be regulated with the same architecture. In this case the autoregulation plays two roles: it enhances the positive feedback loop for the stem cell box; it also decreases the bandwidth of the amplitude filter thereby providing a sharper cutoff.

### Predictions

We now discuss several predictions that emerge from this model.

**Figure 10:**
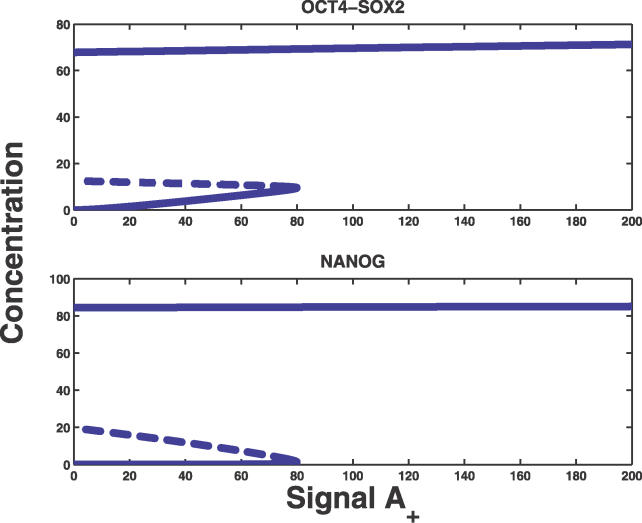
Steady State Concentrations of OCT4–SOX2 and NANOG as Functions of the Signal *A*_+_ There is only one turning point leading to an irreversible bistable switch. At the threshold *A*_+_ *≈* 80, the system switches on and remains on even when the stimulus is removed.

**Figure 11:**
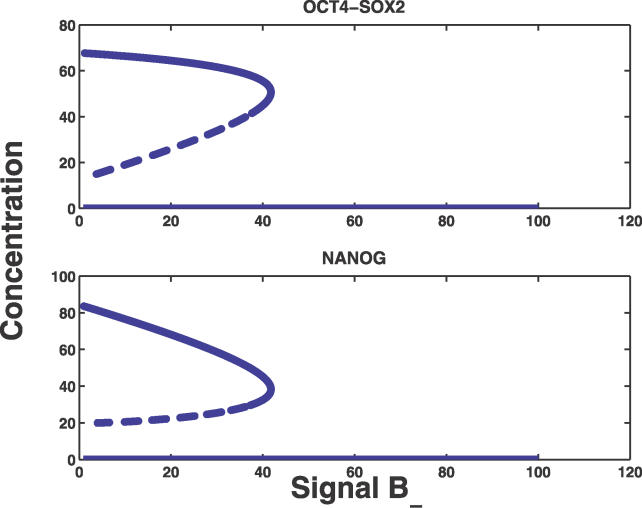
Steady State Concentrations of OCT4–SOX2 and NANOG as Functions of the Signal *B*_−_ At the threshold *B ≈* 41 the system switches off and remains permanently off even when *B*_−_ is removed.

**Figure 12:**
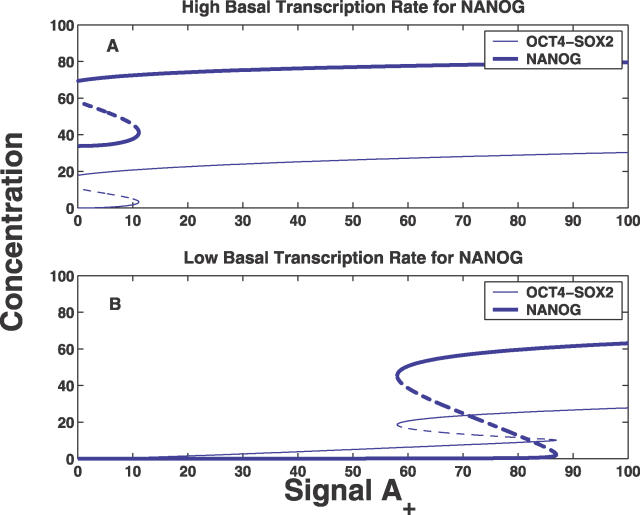
The Effects of Overexpression of NANOG on the OCT4–SOX2 and NANOG Concentration (A) Shows the OCT4–SOX2 and NANOG concentration showing irreversible bistability, when NANOG is overexpressed through a high value for the basal rate of transcription (i.e., *η*_5_). Hence its expression remains on even on removal of the signal *A*_+_. (B) Shows OCT4–SOX2 and NANOG concentrations (the same as in Figure 3) exhibiting bistable behavior, which have negligible basal rates of transcription for NANOG, but which require the signal for the stem cell box to be on.

1) We have explored the parameter space of the model (see Protocol S1) and found that if the binding efficiency of OCT4–SOX2 and NANOG to both OCT4 and SOX2 is increased, one obtains an irreversible bistable switch. In Figures 10 and 11 the OCT4–SOX2 and NANOG concentrations are shown as functions of the signals *A*_+_ and *B*_−_, respectively. As compared with the corresponding curves in Figures 3 and 4, the irreversible nature of the switch with such binding efficiencies is clear. Once the signal *A*_+_ activates the system and the stem cell box is turned on, it remains on even with subsequent removal of the signal. If now a pulse of *B*_−_ is applied, NANOG will be repressed, and its feedback to OCT4 and SOX2 will shut down the stem cell box. On removal of *B*_−_, the stem cell does not go back to a stem cell state, but instead differentiates. One is therefore able to obtain differentiated cells “on demand.”

2) Overexpression of NANOG has recently been shown to propagate the self-renewal of human ES cells over several generations [50], even on reduction of the signal *A*_+_. Overexpression of NANOG can be interpreted in our model as a highly leaky transcription rate of NANOG (i.e*.*, high values for *η*_5_ in Equation 1. In Figure 12A, the OCT4–SOX2 and NANOG concentrations exhibit irreversible bistability. When NANOG is overexpressed it binds to OCT4 and SOX2 and turns on the stem cell box. Even when the signal *A*_+_ is removed, NANOG basal rates are so high that it continues to provide the impetus for the switch. In comparison, Figure 12B shows reversible bistable behavior (the same as Figure 3), for which negligible basal rates of transcription for NANOG have been chosen, but for which the signal *A*_+_ is required for the stem cell box to be on.

3) The architecture of the feedforward loop governing the TGs is consistent with the loss of expression of stem cell TGs and expression of differentiation TGs on suppression of OCT4–SOX2, e.g., by reducing the signal *A*_+_. One can further test whether the feedforward regulation of the stem cell TGs is incoherent or coherent by increasing the binding efficiency of NANOG to the TGs. This results in decreasing/increasing expression of the stem cell TGs for options I and II, respectively.

4) Analogous to the above case, decreasing OCT4–SOX2 has different effects on the differentiation TGs, depending on the type of architecture which applies: with option I, decreasing OCT4–SOX2, from a high level, at which the differentiation genes are repressed, first leads to an increase in expression of the differentiation TGs, as the OCT4–SOX2 level passes through the maximum of the amplitude filter curve (see Figure 7A), then passes through the linear region, and finally decreases to zero. With option II, the gene expression jumps to a high value and thereafter remains almost constant (Figure 8B). These two predictions require that the expression levels of the TGs be measured as a function of the dosage of the signal *A*_+_. Perhaps a more favorable option would be to measure the above with respect to a signal which shuts off NANOG, similar to the effects of p53 as seen in mouse ES cells.

The model suffers from a few deficiencies. Overexpression of OCT4 initiates differentiation [1,51,52]; similarly, expression of NANOG initiates differentiation in mouse [50], and this is not taken into account into our model. Also, it is likely that the target stem cell genes are also involved in transcriptional control of OCT4, SOX2, and NANOG [53]. Given that OCT4 also binds to some downstream genes individually, not always together with the SOX2 and NANOG [3,4], one can easily imagine mechanisms to account for what is observed in [1,51,52]. However, there is insufficient knowledge available for a falsifiable extension of the model to explain this effect.

Future work will elaborate on the effects of stochastic fluctuations in protein concentration of NANOG, OCT4, and SOX2 on the regulatory behavior of the TGs. Bistability is one way to counter chatter in input noise [54], and hence we expect that the bistable behavior of the stem cell box should be robust to input environmental fluctuations compared with the case where it is ultrasensitive [55]. Preliminary analysis of the incoherent feedforward nature of the regulation shows that along with the amplitude filtering of a signal, noise in the OCT4–SOX2 input as well as NANOG fluctuations contribute significantly to the TG output noise. It is therefore likely that TGs must have significant feedback mechanisms to suppress the noise, but yet to be responsive to the signal. Finally, recent computational modeling [56] has shown how it is possible to combine single cell behavior to population dynamics by studying effects of external factors. A better understanding of the core transcriptional dynamics will aid in such population studies, thereby etching out a clearer picture of pathways that control stem cell self-renewal/pluripotency and differentiation.

## Materials and Methods

**Figure 13:**
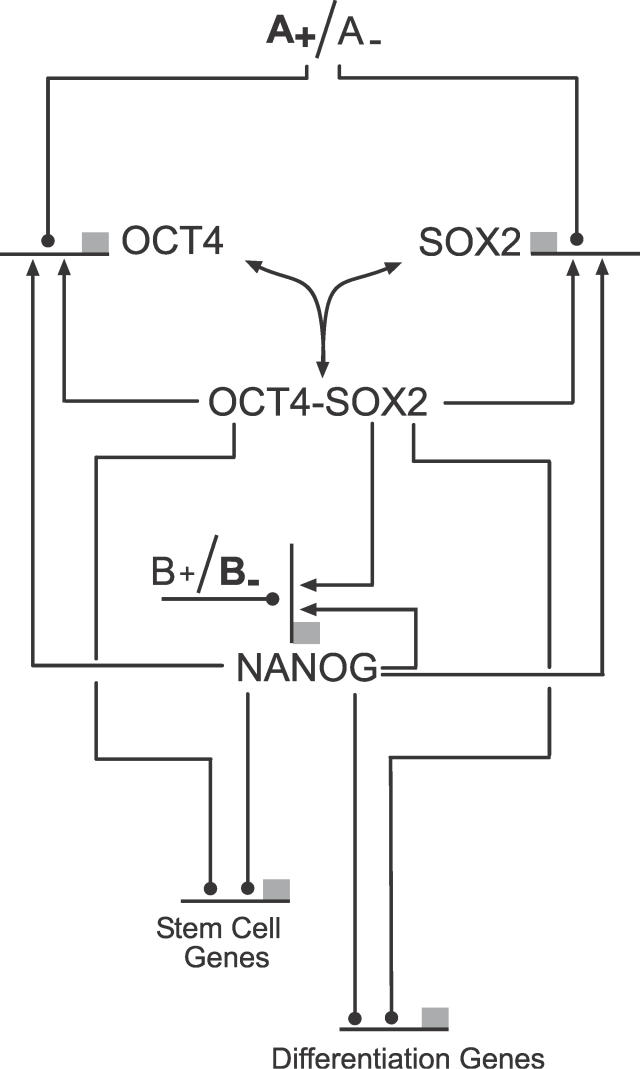
Stoichiometric Map of the Model Corresponding to Figure 1 All protein species are assumed to have a first-order degradation rate. The lines ending with dots could be either activating or repressing, depending on the choice of model.

A stoichiometric map for more detailed model considerations corresponding to Figure 1 is shown in Figure 13. SBML models for this map are available at http://www.sys-bio.org.

### Derivation of transcription rates.

We use rate equation models, which are deterministic and deal with molecular concentrations rather than with individual molecules. For small molecule numbers, one can use Monte Carlo simulations of the Master Equation [57]. However, in our case, it appears that the number of transcripts involved is about 50 to 100 [27], which should be a sufficient number for a deterministic approach. A commonly used approach for deterministic models is to use Michaelis–Menten equations or variants thereof, e.g., Hill equations. We follow the Shea–Ackers formalism [16], described in detail for several kinds of gene regulation in [18–20]. The advantage here is the transparency of the relative importance of the different interactions between TFs and RNAP and the close relationship between the theory and the experiment. We explicitly consider the binding/absence of TF and RNAP at the gene promoter region, such that it is easy to convert the combinatorial approach taken here to a particular reaction scheme. We re-derive this well-known result for the sake of completeness in Protocol S1.

We assume that the process of binding/unbinding of TFs and RNAP takes place over a time scale much smaller than the rate of change of the concentrations of all the proteins in the network. This corresponds to thermodynamic equilibrium at the promoter region of each gene. The transcription rate is then given by the fraction of cases when the RNAP is bound. We compute the partition function [18] by enumerating all the possible cases by which the different combinations of proteins bind to the gene. For the OCT4 and SOX2 genes, we assume four binding sites corresponding to (i) the signal TF (*A*_+_), (ii) the heterodimer OCT4–SOX2 (*OS*), (iii) NANOG (*N*), and (iv) the RNAP enzyme (*R*). There are sixteen possible combinations by which these four proteins can bind to the DNA. The same holds for NANOG, where in addition to OCT4–SOX2, NANOG, and RNAP, we also assume the existence of a signal *B*_−_, which has the effect of suppressing NANOG (similar to the effect that p53 has on mouse ESC). We model the stem cell and differentiating downstream TGs as being regulated by OCT4–SOX2, NANOG, and RNAP. Hence they have three binding sites and eight possible combinations by which various combinations of proteins can bind. For each such combination there is an associated free energy of binding δ*G*. The transcription rate is proportional to the relative probability that the polymerase is bound to the gene. This probability can be computed using the partition function, which for OCT4 and SOX2 regulation reads

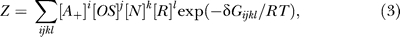
where *i*, *j*, *k*, *l* = (0,1) corresponds to unoccupied/occupied binding sites, respectively, and where δ*G_ijkl_* is the corresponding free energy, and *T* and *R* are the temperature and gas constant, respectively. If the TF is an activator, its free energy to bind in combination with the RNAP is very low, and hence it is favored. In contrast, for an inhibitor the opposite is true, and hence it is unlikely that a repressor AND RNAP will bind. The cooperativity, or in some cases the repulsion between proteins, is what generates the regulatory rules. If *Z*_on_ and Z_off_ represent the partial sums for the cases where the DNA is bound and not bound, respectively, then the transcription rate is proportional to

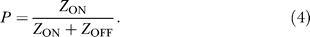



### Regulation of OCT4, SOX2, and NANOG.

We model the stem cell box using the formalism defined by Equations 3 and 4. For both *Z*_on_ and *Z*_off_ in Equation 4 the number of terms grows exponentially with the number of players. For example, with three TFs and RNAP, one has eight terms in each of them, leading to sixteen parameters for the free energies. If one has access to sufficient dynamical measurements in terms of time series, one could in principle determine the parameters. For the ES cell switch, this is not the case. However, with some information about the nature of the interactions and the requirement of switch behavior, it is natural to assume that the interactions are dominated by a few terms and to evaluate the consequences of the assumption including features such as robustness. More specifically we assume that NANOG cannot bind to the OCT4 and SOX2, unless recruited by the OCT4–SOX2 complex. Also it is reasonable to assume that the signal *A*_+_ is only effective when the promoter region is not occupied by the OCT4–SOX2, or the OCT4–SOX2 together with NANOG. Furthermore, we assume that *A*_+_, OCT4–SOX2–NANOG, and OCT4–SOX2 are activators. We make a modeling simplification by assuming that the OCT4–SOX2 heterodimerization occurs before binding to the OCT4, SOX2, NANOG, and TGs. A more detailed model—which assumes that OCT4 first binds to the DNA, recruits SOX2, and then this further recruits either RNAP, or NANOG, the latter as a repressor—yields qualitatively the same type of dynamics. Hence the model we describe deals with the OCT4–SOX2 complex as an input to OCT4, SOX2, NANOG, and the TGs. For the OCT4 and SOX2 genes, the emerging “truth table” is shown in Tables 1 and 2. The corresponding rates of occupancy of the promoters are proportional to

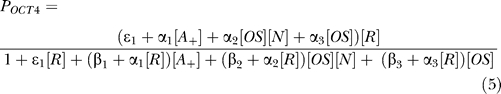
and

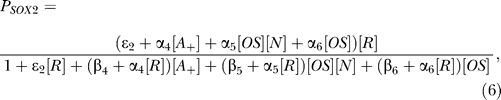
respectively. The parameters *α_i_* and *β_i_* are related to the free energies for the cases when RNAP binds and does not bind, respectively. In Equations 5 and 6, and Table 1, as well as in subsequent equations for the RNAP occupancies, the terms which only depend upon [*R*], for example *ɛ*_1_[*R*] and *ɛ*_2_[*R*], represent leaky transcription; small probabilities are assigned for the RNAP to bind to the gene, even in the absence of any activators. The occupancies of Equations 5 and 6 are converted into a transcription rate, by multiplying it by a numerical constant, which can be regarded as an average rate of transcription.

For the NANOG gene, we assume that NANOG cannot bind unless recruited by the OCT4–SOX2 complex. Also, the signal *B*_−_ can act only when the promoter region is unoccupied by either the OCT4–SOX2 complex or the OCT4–SOX2–NANOG complex. B acts as a repressor, whereas OCT4–SOX2 and OCT4–SOX2–NANOG are activators. The rate of occupancy of the promoter (see Table 2), is therefore proportional to

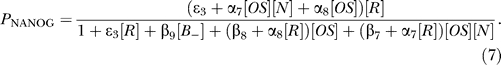



The functional form of the transcription rates used in Equation 1 follows directly from Equations 5–7.

### Regulation of TGs.

For the downstream TGs, which are co-regulated by OCT4–SOX2 and NANOG, we explore two different types of regulation, cases I and II, respectively (see earlier text). In case I the same incoherent architecture is shared for the stem cell and differentiation TGs. For the latter, the scheme used is that the OCT4–SOX2 complex is an activator. However, the OCT4–SOX2 complex recruits NANOG, and together this complex acts as a repressor. The other two possibilities are: for the stem cell genes both OCT4–SOX2 and NANOG are activators, and the last case, for the differentiation TGs both OCT4–SOX2 and NANOG are repressors (case II).

In Table 3 we show the various truth tables for the TGs. From Table 3A, we obtain the regulation with common architecture for the stem cell TGs, which uses the incoherent feedforward structure.

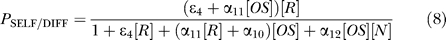



The functional form of the transcription rate used in Equation 2 follows from the above. With the assumption that both OCT4–SOX2 and NANOG are activators, we obtain for the regulation of the stem cell TGs from Table 3B:

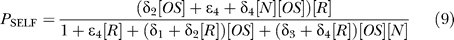



Finally, for the last option for the differentiation TGs, for which both OCT4–SOX2 and NANOG are repressors, we obtain from Table 3C:

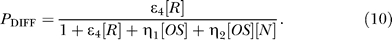



### Network subgraph searches.

The algorithm used to search for specific subgraphs in the model organism transcriptional networks is similar to that described in [22]. All *n*-node subgraphs are enumerated, and based on the interactions between the constituent genes, each subgraph is expressed in a canonical form with respect to permutations of the *n* labels. Finally, all but the subgraphs of interest are filtered out.

Reducing the network in Figure 1 to pure gene–gene interactions yields a maximally connected three-node directed network as shown in Figure 2A. We searched for occurrences of this network in the E. coli transcriptional network from the Web site related to [22], the S. cerevisiae transcriptional network of the yeast proteome database [23], and the D. melanogaster network derived from GeNet [24]. However, none of these searches resulted in any matches. In fact, the E. coli network does not contain any pairs of mutually regulating genes, and we therefore disregard it for most of this discussion. This apparent lack of mutual regulation may seem strange, but recall that we only consider interactions at the transcriptional level. Protein–protein interactions play a complementary role [58] and might be particularly important in small, fast-paced organisms such as E. coli.

Widening our search by removing autoregulations (Figure 2B) does not make any difference for the yeast network, but in *Drosophila* we then find a single match. This match corresponds to Krüppel, Knirps, and Giant, three genes involved in early embryonic development. Although structurally similar (except for the autoregulation), the OCT4/SOX2/NANOG and Krüppel/Knirps/Giant subnetworks contain radically different interactions (see Results).

In a further attempt to find architectural elements similar to the stem cell box, we searched for pairs of mutually activating genes, preferably also activating themselves (Figure 2C). This subgraph would correspond to a minimal version of the stem cell switch, without the redundancy of having both OCT4 and SOX2. The closest match in the yeast network consists of CAT5 and CAT8, which act as activators for each other. Additionally, CAT5 activates itself. In *Drosophila* the same relationship exists between achaete and scute. We also note that the human hematopoietic switch consisting of the genes *PU.1* and *GATA1* looks much like Figure 2C, in which *PU.1* and *GATA1* upregulate themselves, but repress each other, essentially resulting in a bistable switch, which separates two differentiation paths in hematopoietic stem cells [59]. The symmetry between the two final states here contrasts to the asymmetry between the two states of the stem cell box.

Finally, since a series of two repressions in some sense amounts to a positive regulation, we expanded the search to include the motif shown in Figure 2D, where the presence of A prevents B from repressing C, thus making C follow A. No instances of this type of mutual activation were found in yeast, but in the *Drosophila* dataset it appeared three times, involving the genes *engrailed* and *hedgehog*. However, these two genes are highly connected in the regulatory network, and many other genes could affect the would-be switches to the point where their behavior is far different from that of a simple bistable switch.

### Simulations.

All simulations were carried out using the Systems Biology Workbench (SBW/BioSPICE) tools [60]: the network designer JDesigner, the simulation engine Jarnac [61]. Bifurcation diagrams were computed using SBW with an interface to MATLAB [62], and a bifurcation discovery tool [63]. Bifurcation plots were also computed and cross-checked using Oscill8 (http://sourceforge.net/projects/oscill8), an interactive bifurcation software package which is linked to AUTO [64] and SBW [60]. In all our simulations the species concentrations are regarded as dimensionless, whereas the kinetic constants have dimensions of inverse time, with dimensionless Michaelis–Menten constants. All models are available as standard SBML or Jarnac scripts.

## Supporting Information

Protocol S1 Supplementary Text (276 KB PDF)

## Abbreviations

ES: embryonic stem cells
RNAP: RNA polymerase
TF: transcription factor
TG: target gene
