# Supplementary material for: Transcriptional Dynamics of the Embryonic Stem Cell Switch
Source: PLoS Comput Biol. 2006 Sep 15;2(9):e123. doi: 10.1371/journal.pcbi.0020123 (PMC1570179; doi:10.1371/journal.pcbi.0020123)
Supplement: Supplementary file 1 [file pcbi.0020123.sd001.pdf]

Supplementary Text

Transcriptional Dynamics of the Embryonic Stem Cell Switch

V. Chickarmane, C. Troein, U. Nuber, H. M. Sauro and C. Peterson

Transcription rates from an equilibrium calculation

We rederive the well known result that an equilibrium calculation, which is commonly used in enzymatic reactions, yields the same transcription rate as the statistical mechanics approach [1] we use. We show that this approach not only leads to useful insight about the model but can also be used to compare directly to experimental results, since the model uses measurable equilibrium reaction rates. We illustrate this by first evaluating the transcription rate using the statistical mechanics approach and then comparing to the equilibrium approach for a simple system. Consider an example with a single gene that is autoregulatory, *i.e.* the transcription rate is enhanced when it is bound by its own protein product, X. There are then four possible states of the system. With the logic shown in Table S1, the transcription rate is given by

$$T \propto \frac{(\delta_1 + \delta_2[X])[R]}{1 + \delta_1[R] + (\delta_3 + \delta_2[R])[X]} \tag{S1}$$

In Eq. (S1), δ_i are related to the binding free energies, and $[R]$ is the RNAP concentration. Denote by G_0, G_X, G_R and G_{XR} the four states of the gene; unbound, bound by the transcription factor X, bound by the RNAP and bound by both X and RNAP. These states are subject to a normalization

$$G_0 + G_X + G_R + G_{XR} = 1 \tag{S2}$$

The following reaction scheme defines the network

$$\begin{aligned} X + G_0 &\underset{k_{1b}}{\overset{k_{1f}}{\rightleftharpoons}} G_X \\ R + G_0 &\underset{k_{2b}}{\overset{k_{2f}}{\rightleftharpoons}} G_R \\ R + G_X &\underset{k_{3b}}{\overset{k_{3f}}{\rightleftharpoons}} G_{XR} \end{aligned} \tag{S3}$$

At thermodynamic equilibrium, one has

$$K_1 = \frac{[G_X]}{[G_0][X]} \quad K_2 = \frac{[G_R]}{[G_0][R]} \quad K_3 = \frac{[G_{XR}]}{[G_X][R]} \tag{S4}$$

where $K_i = k_{if}/k_{ib}$ are the equilibrium constants. From Eqs. (S3,S4) and Eq. (S2), we compute the fractional probability P of the gene being bound by RNAP,

$$P \propto [G_R] + [G_{XR}] = \frac{K_2[R] + K_1K_3[R][X]}{1 + K_2[R] + K_1[X] + K_1K_3[X][R]} \tag{S5}$$

which is functionally similar in form to Eq. (S1). Both methods are equivalent as the only physical requirement is thermal equilibrium for these reactions. Although the statistical mechanics approach is more intuitive, the equilibrium approach allows us to define a reaction scheme, which can be described in terms of measurable kinetic constants. It is also clear that such a scheme is required to carry out a stochastic simulation.

Parameter dependence of the stem cell box switch behavior

Next we investigate how the model parameters determine the dynamics of the stem cell box. More precisely, we probe the parameter range for which one obtains a switch-like behavior. In other words, is the switch-like behavior robust? The curves of the steady state values of the concentrations of OCT4, SOX2, NANOG and the OCT4–SOX2 complex in Figs. 3 and 4 in the main text as functions of the environmental factor A_+ and signal B_- exhibit bistable

switch-like behavior with two saddle-node bifurcations (SN). These curves were obtained using the parameter sets given in the Table S2. We study the parameter dependence by asking as to how the switch-like behavior changes as the parameters around the nominal parameter set displayed in Table S2 are varied. Thus, we follow the SN of the bifurcations in Fig. 3 as functions of the various parameters. In effect, these are two-parameter bifurcation plots. We therefore plot the locus of the thresholds of the environmental factor A_{+}^{0} , at which the switch is turned ON, as a function of the parameters. In each of the plots, that we will subsequently display (Figs. S1-S4) the y-axis represents the particular parameter value being varied, and the x-axis the thresholds A_{+}^{0} at which the SN occurs. We obtain either of two cases: For each parameter value, there are two points corresponding to a bistable switch with two turning points or a single threshold corresponding to an irreversible bistable switch. If the threshold value is zero, which happens for extremely low parameter values, it corresponds to the disappearance of the switch-like behavior altogether.

Binding strengths of the OCT4–SOX2 and OCT4–SOX2–NANOG complexes. In studying the parameter dependence of the binding strengths of the protein complexes on the three genes, we use the fact that the nature of the regulation on OCT4 and SOX2 is similar. Hence, it is sufficient to study the parameter dependence of the binding at the NANOG gene and either of the OCT4 and SOX2 genes. We therefore consider binding strengths a_3 and a_2 which determine how strongly OCT4–SOX2–NANOG and OCT4–SOX2 bind to OCT4, respectively, and e_2 and e_1, the binding strengths of the OCT4–SOX2–NANOG and OCT4–SOX2 complexes on the NANOG gene respectively (see Eq. (1) in the main text. From Fig. S1, one observes the threshold at which the switch-like behavior occurs reduces as the free energy of binding decreases, or as a_3, a_2, e_2 and e_1 increase. This is intuitively obvious, since the stronger that these complexes bind to OCT4, SOX2 and NANOG, the higher the transcription rate, and hence a smaller threshold of the environmental factor is required to turn the switch ON. Although qualitatively similar results are obtained for the binding strengths of the OCT4–SOX2–NANOG and OCT4–SOX2 complexes on OCT4 and

NANOG, the threshold A_+^0 of the environmental factor, which is required to turn the switch ON, is much smaller for the NANOG gene, as compared to the binding at OCT4 and SOX2. This suggests that the OCT4–SOX2–NANOG complex binding to NANOG, can be much weaker as compared to its binding at OCT4 and SOX2, and yet have significant effect on the dynamics. This is because the regulation at NANOG is autoregulatory which turns out to be crucial in kick-starting the stem cell box switch. As can be seen From fig. S1, the values for a_3, a_2, e_2 and e_1 range over almost three orders of magnitude, *i.e.* from .01 to 10, for which the switch-like behavior is exhibited[1]. Hence, the switch is extremely robust.

There is an additional interesting feature in the behavior of a_3, e_2, the binding strengths of the OCT4–SOX2–NANOG complex on the OCT4 (SOX2) and NANOG genes respectively. From Fig. S1, when $a_3 > 0.4$ and $e_2 > 0.15$ there is only one turning point. This means that the switch is irreversible for this range of parameters. Hence in this situation, if the environmental factor A_+, is initially turned on to a large value, the stem cell box is turned ON, but if it is removed, the switch remains ON. The irreversible switch is however only possible if the OCT4–SOX2–NANOG complex binds strongly to OCT4 (SOX2) and NANOG. It is therefore interesting to speculate that if this binding strength could be tuned with mutational techniques, *i.e.* to vary between strong and weak, then the irreversible switch could be turned into a reversible one and vice versa.

Formation and degradation of the OCT4–SOX2 complex. In Fig. S2 we show the formation, dissociation and degradation rates, k_{1c}, k_{2c} and k_{3c} respectively, of the OCT4–SOX2 complex as functions the threshold A_+^0, at which the switch is turned ON. As k_{2c} and k_{3c} increase, A_+^0 also increases since the SOX2 and OCT4 transcription rates must be increased to account for loss of the OCT4–SOX2 complex. The opposite effect occurs for k_{1c}, since as it increases, the rate of complex formation is increased and hence a lower threshold is required to turn the switch ON. For $k_{1c} > 0.5$, the system becomes a irreversible switch.

[1] There is strictly only a lower limit, since if the complexes cannot bind to the genes, no regulation occurs.

Degradation rates of OCT4, SOX2 and NANOG proteins. As can be seen from Fig. S3, increasing the degradation rates of OCT4, SOX2 and NANOG effectively increase the threshold of the switch, since a higher activation from the environmental factor is required to account for loss of proteins through degradation. Degradation of the NANOG protein is particularly sensitive in that the threshold dramatically increases on increase of its degradation rate. The switch-like behavior is lost when the degradation rate of NANOG reaches $g_3 > 1.5$ and degradation rate for OCT4/SOX2 reaches $g_1 > 5$. The degradation rate of NANOG is much more sensitive to any of the other parameters due to its strong impact upon the dynamics. Being autoregulatory, the degradation rate of NANOG is crucial in determining its steady state value.

Basal transcription rates of the OCT4, SOX2 and NANOG genes. The basal transcription rates have the effect of reducing the threshold of activation of the switch, since transcription occurs even for a smaller environmental factor A_+. Hence, as can be seen from Fig. S4, A_+^0 decreases with increased basal rates. Also deduced from Fig. S4 is the relative importance of the basal transcription rate η_5 for the NANOG gene. Again the autoregulatory nature of NANOG requires a lower basal transcriptional rate to turn ON the switch.

Parameter values for Figures 10, 11 in the main text. The same parameters as in Table S2 are used to generate Figs. 10 and 11 in the main text except for the changes shown in Table S3. To obtain Fig. 11 we first assume that the stem cell box is ON (steady state values of all the species for $A_+ = 100$). A is then set to zero (removal of the environmental factor) and the steady states of the system are evaluated as function of signal B_-.

Equations and Parameters for Target Gene Regulation

To study the dynamics of the target genes, due to regulation by OCT4–SOX2 and NANOG, we initially ignore the feedback from NANOG to OCT4 and SOX2. Hence, we assume the input to the system, OCT4–SOX2, to be constant. The equations determining the regulation

of the target gene product are then given by

$$\frac{d[N]}{dt} = \frac{(\eta_5 + e_1\ [OS] + e_2\ [OS]\ [N])}{(1 + \eta_6 + f_1\ [OS] + f_2\ [OS]\ [N] + f_3\ [B])} - \gamma_3[N], \tag{S6}$$
$$\frac{d[TG]}{dt} = \frac{\eta_7 + g_1\ [OS]}{1 + \eta_8 + h_1\ [OS] + h_2\ [OS][N]} - \gamma_4[TG],$$

where $[TG]$ refers to the concentration of the target gene product. The parameters used for Fig. 6 are given in Table S4 (with $f_3 = 0$). The curves in Fig. 6, which refer to the simple feedforward network where NANOG does not have autoregulation are obtained by simulating the above equations for $e_2 = 0$, $f_2 = 0$, and with the same values as in Table S4, for the remaining parameters. In Fig. 6C the expression for the self-renewal gene is plotted for the parameters in Table S4, with the change, $h_2 = 0.001$.

The equations, which are used to describe the regulation of the self-renewal target genes (TG) with OCT4–SOX2 and NANOG as activators and differentiation TG's with OCT4–SOX2 and NANOG as repressors, are given below. These follow from the truth table given in Table 3B and Table 3C respectively.

$$\frac{d[TG]_{self-renewal}}{dt} = \frac{\eta_9 + m_1\ [OS] + m_2[OS][N]}{1 + \eta_{10} + n_1\ [OS] + n_2\ [OS][N]} - \gamma_5[TG]_{self-renewal}, \tag{S7}$$
$$\frac{d[TG]_{differentiation}}{dt} = \frac{\eta_{11}}{1 + \eta_{12} + q_1\ [OS] + q_2\ [OS][N]} - \gamma_6[TG]_{differentiation},$$

The above equations are used in conjunction with the equations for the stem cell box, to generate the curves in Fig. 8 in the main text.

Parameters for Integrated Model

In the integrated model we combine Eqs. (1,2), from which we generate Fig. 7 in the main text. NANOG now feeds back to OCT4 and SOX2. For the combined model the parameters for the OCT4, SOX2 and NANOG genes are the same as in Table S2. The target gene

parameters are given in Table S5. For Fig. 7B in the main text, which shows the output of self-renewal genes, the parameter $h_2 = 0.001$ is used, and the remaining parameters are the same as in Table S5.

For Fig. 8, in which we study the dependence of the self-renewal (Fig. 8A), and differentiation (Fig. 8B) TG products as a function of the input environmental factor A_+, we use the parameters given in Table S6 (these correspond to Eq. S7.). The target gene parameters are given in Table S5.

The parameters used to generate Fig. 9 are the following: The target gene parameters for all sets of curves are given in Table S5. For Fig. 9A, the stem cell box parameters are the same as in Table S1, except for the changes given in Table S7. For Fig. 9B, which displays the TG expression for strong binding of NANOG to the OCT4 and SOX2 genes, we use the parameters given in Table S8. The only parameter which is changed is $h_2 = 0.01$.

For Fig. 12, which shows the irreversible bistability for NANOG over-expression, we use the same parameters as in Table S2, except for the following changes, $\eta_5 = 35, \eta_6 = 0.035$. The target gene parameters are given in Table S5.

In all of the figures generated which are referred to in this section the parameter $f_3 = 0$.

Dependence of Target Gene expression assuming an incoherent feedforward architecture.

Next we comment on the dependence of the target gene expression assuming an incoherent feedforward architecture, on network parameters. The motivation is to study the response of the amplitude filter curve as a function of the binding strengths of the transcription factors at the target gene. We use the parameters given in S4. From Eq. (2) in the main text, for the rate of change of the target gene product, the two key parameters are g_1, h_2, the strength of binding of the OCT4–SOX2 complex, and the OCT4–SOX2–NANOG complex to the target genes, respectively. We noted earlier that as the free energy of binding of

the OCT4–SOX2–NANOG to the target gene increases, or as $h2$ decreases, the target gene gets increasingly expressed. This is seen in the third panel in Fig. S5, which shows that as h_2 decreases, the target gene repression increases and we loose the sharp cutoff of the steady state value of the protein level with respect to OCT4–SOX2 protein levels. In the middle panel, g_1, is varied, which determines the rate of transcription of the target gene. Increasing the amount of the input OCT4–SOX2 complex has the effect of simply increasing the total amount of target gene protein level. Finally, in the uppermost panel, we vary e_2, the binding affinity of the OCT4–SOX2–NANOG complex to the NANOG gene, which essentially tunes the amount of autoregulation. Decreasing the amount of autoregulation leads to less expression of NANOG, and hence less repression of the target genes.

Tables

X	R	Rate
0	0	1
1	0	δ_3[X]
0	1	δ_1 [R]
1	1	δ_2[R][X]

Table S1: Logics and rates for a single autoregulatory gene.

η_1	a_1	a_2	a_3	η_2	b_1	b_2	b_3	η_3	c_1	c_2	c_3	η_4
10^{-4}	1	0.01	0.2	10^{-7}	0.0011	0.001	0.0007	10^{-4}	1	0.01	0.2	10^{-7}
d_1	d_2	d_3	η_5	e_1	e_2	η_6	f_1	f_2	f_3	k_{1c}	k_{2c}	k_{3c}
0.0011	0.001	0.0007	10^{-4}	0.005	0.1	10^{-7}	0.001	$9.95{\cdot}10^{-4}$	0.01	0.05	0.001	5

Table S2: Parameter values used for Figs. 3 and 4 in the main text. All the parameters used to compute the transcription rates of the genes in the *Materials* section in the main text, *i.e.* free energies and RNAP concentrations ($\alpha_i, [R]$), are converted into the parameters listed in Eqs. (1,2) in the main text. The degradation constants are $\gamma_i = 1, i = 1, 2, 3$.

a_3	c_3	b_3	d_3	e_1	f_2	f_3
0.5	0.5	0.001	0.001	0.01	0.001	0.05

Table S3: Parameters that are changed from the values given in Table S2 when generating Figs. 10 and 11 in the main text.

η_5	e_1	e_2	η_6	f_1	f_2	γ_3
10^{-5}	10^{-4}	10^{-3}	10^{-7}	$9.01 \cdot 10^{-4}$	10^{-3}	0.05
η_7	g_1	η_8	h_1	h_2	γ_4	
10^{-5}	0.1	10^{-7}	0.0019	0.05	10^{-2}	

Table S4: Parameters used in target gene expressions including the NANOG autoregulation.

η_7	g_1	η_8	h_1	h_2	γ_4
10^{-4}	0.1	10^{-7}	10^{-3}	1	0.01

Table S5: Parameters for Fig 7 in the main text.

η_9	m_1	m_2	η_{10}	n_1	n_2	γ_5	η_{11}	η_{12}	q_1	q_2	γ_6
10^{-4}	0.1	0.1	10^{-7}	10^{-3}	10^{-2}	0.05	1	0.001	0.001	0.01	10^{-2}

Table S6: Parameters for Fig 8 in the main text.

a_3	c_3	b_3	d_3	e_2	f_2
0.05	0.05	$5.5 \cdot 10^{-4}$	$5.5 \cdot 10^{-4}$	0.025	$9.25 \cdot 10^{-4}$

Table S7: Parameters for Fig 9A in the main text.

a_3	c_3	b_3	d_3	e_2	f_2
1	1	$1.5\ 10^{-3}$	$1.5 \cdot 10^{-3}$	0.025	$9.25 \cdot 10^{-4}$

Table S8: Parameters for Fig 9B in the main text.

Figures

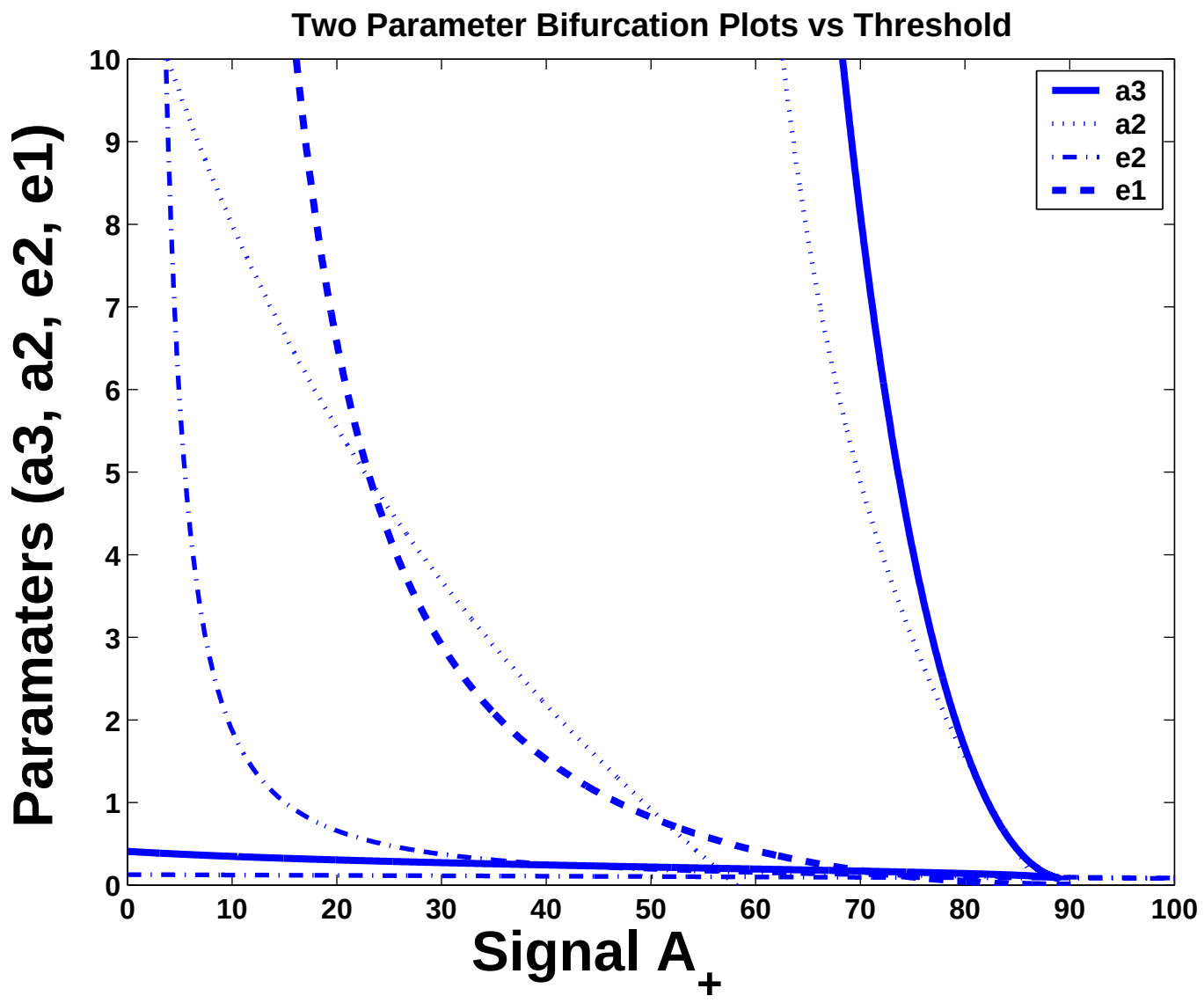

Figure S1: Two parameter bifurcation plots for binding strengths of protein complexes to the genes. The y-axis represents the value of the parameter, in this case a_3, a_2, e_2 and e_1, and the x-axis the threshold A_+^0 of the environmental factor, at which the switch turns ON. For example, if $a_3 = 4.0$, then the turning point would occur at $A_+^0 =\simeq 75$. For low values of $a_3 <= 0.4, e_2 <= .15$, the switch is reversible (there are two turning points). For higher values of a_3, e_2, the switch is irreversible, suggesting that the stem cell box remains in the **ON** state even after removal of the environmental factor A_+.

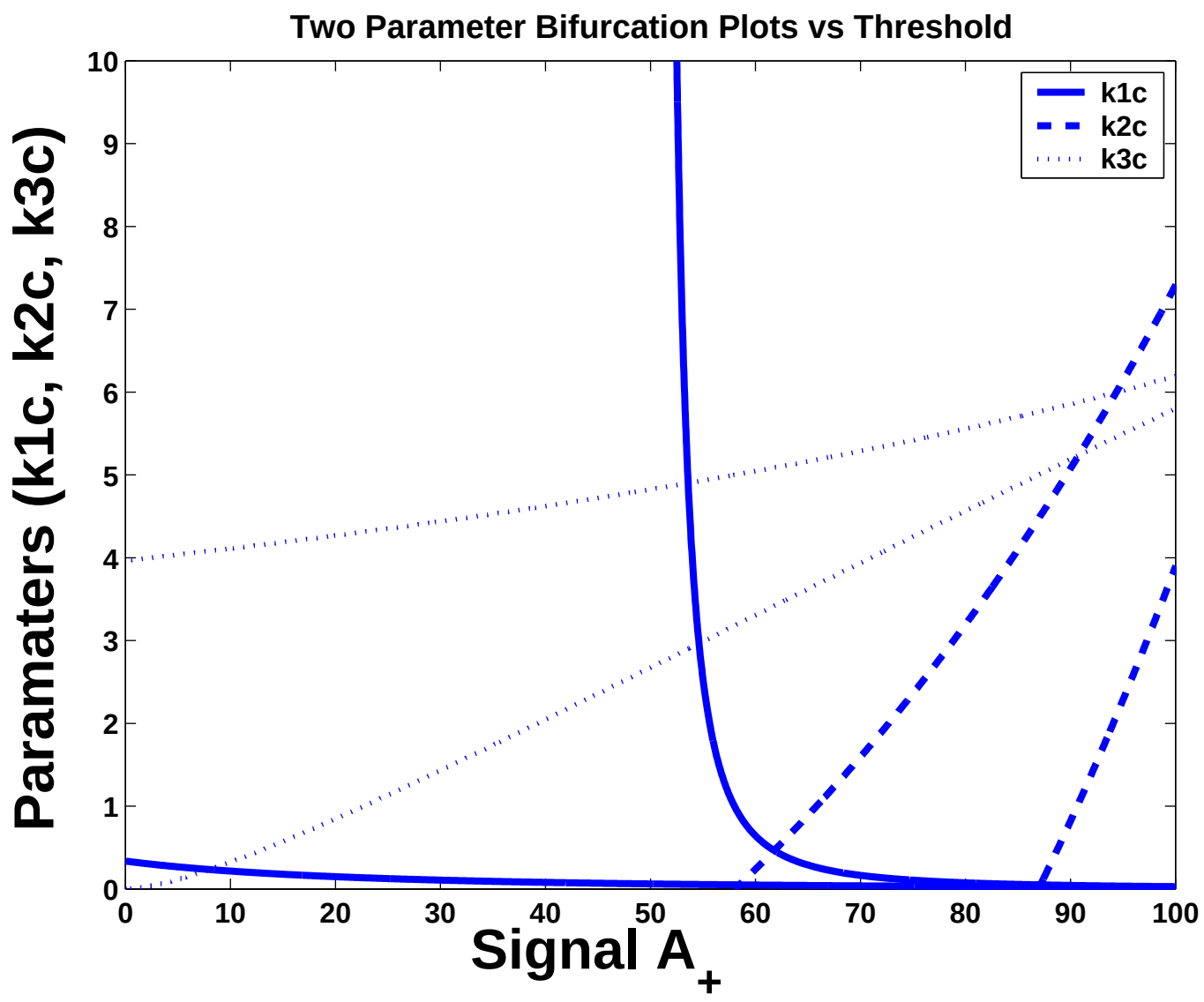

Figure S2: Two parameter bifurcation plots for formation and degradation rates, k_{1c}, k_{2c}, k_{3c} of the OCT4–SOX2 complex. Increasing k_{1c} beyond 0.5, leads to a irreversible switch.

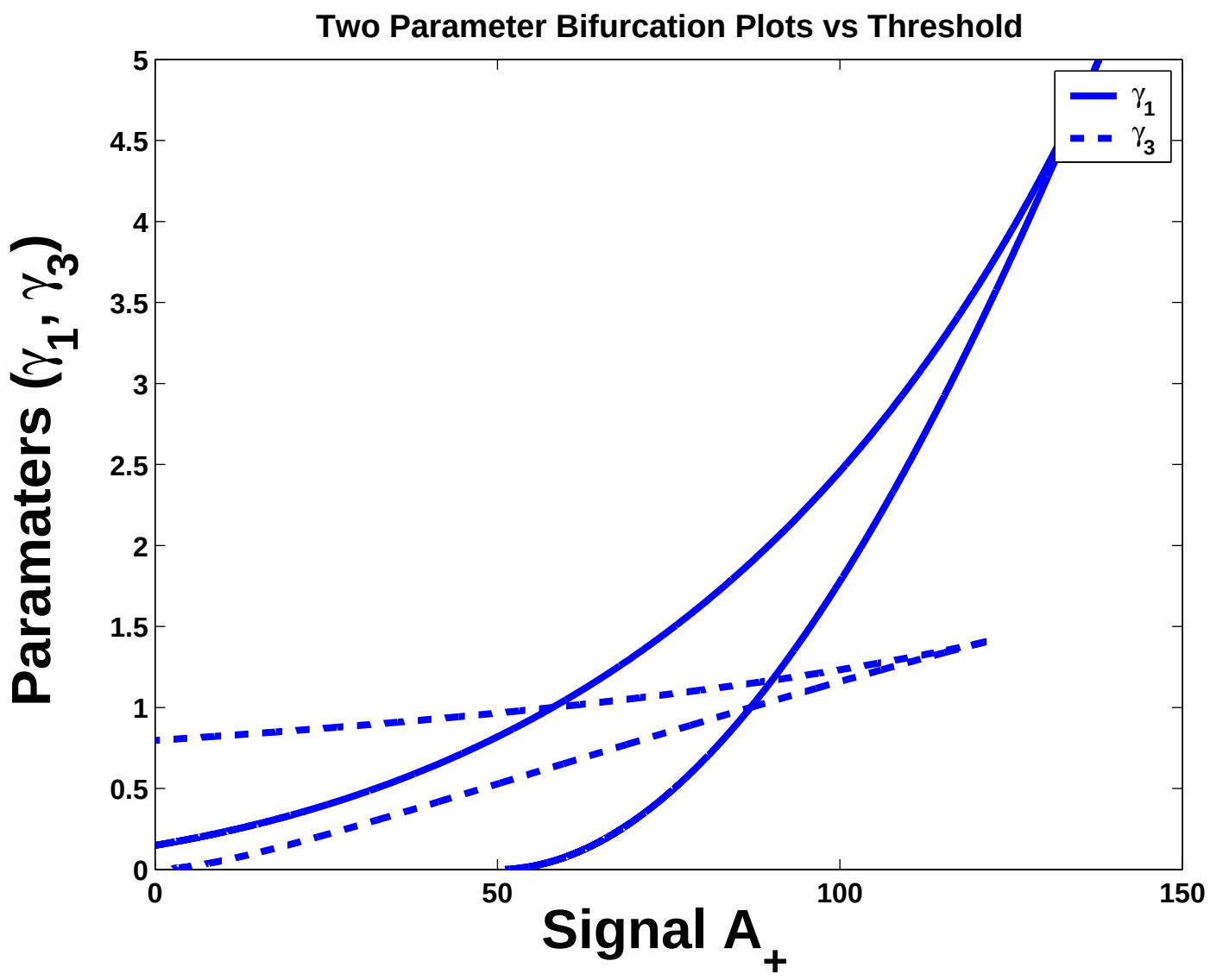

Figure S3: Two parameter bifurcation curves for degradation rates of OCT4, SOX2 and NANOG proteins.

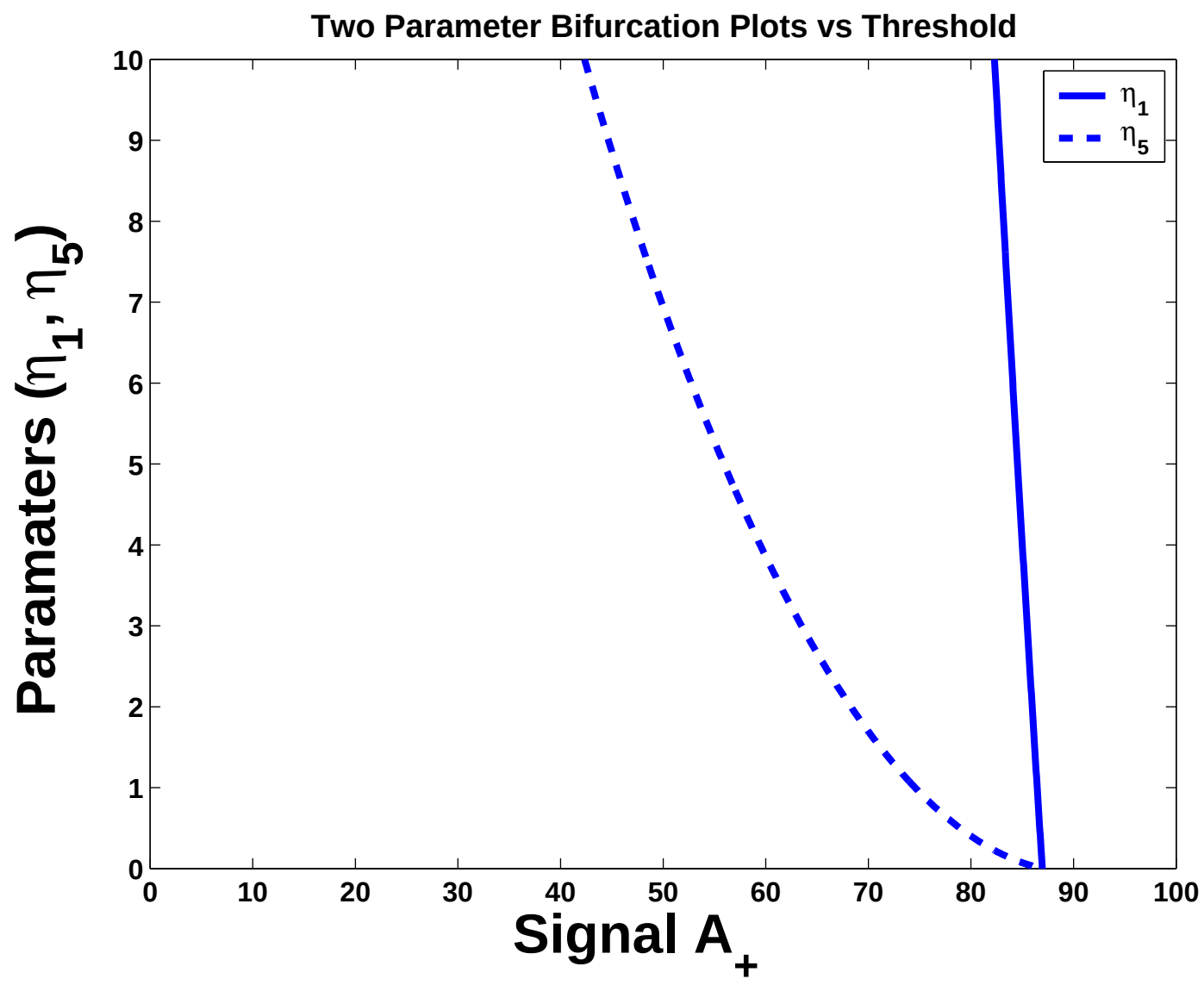

Figure S4: Two parameter bifurcation curves for basal transcription rates of OCT4, SOX2 and NANOG genes.

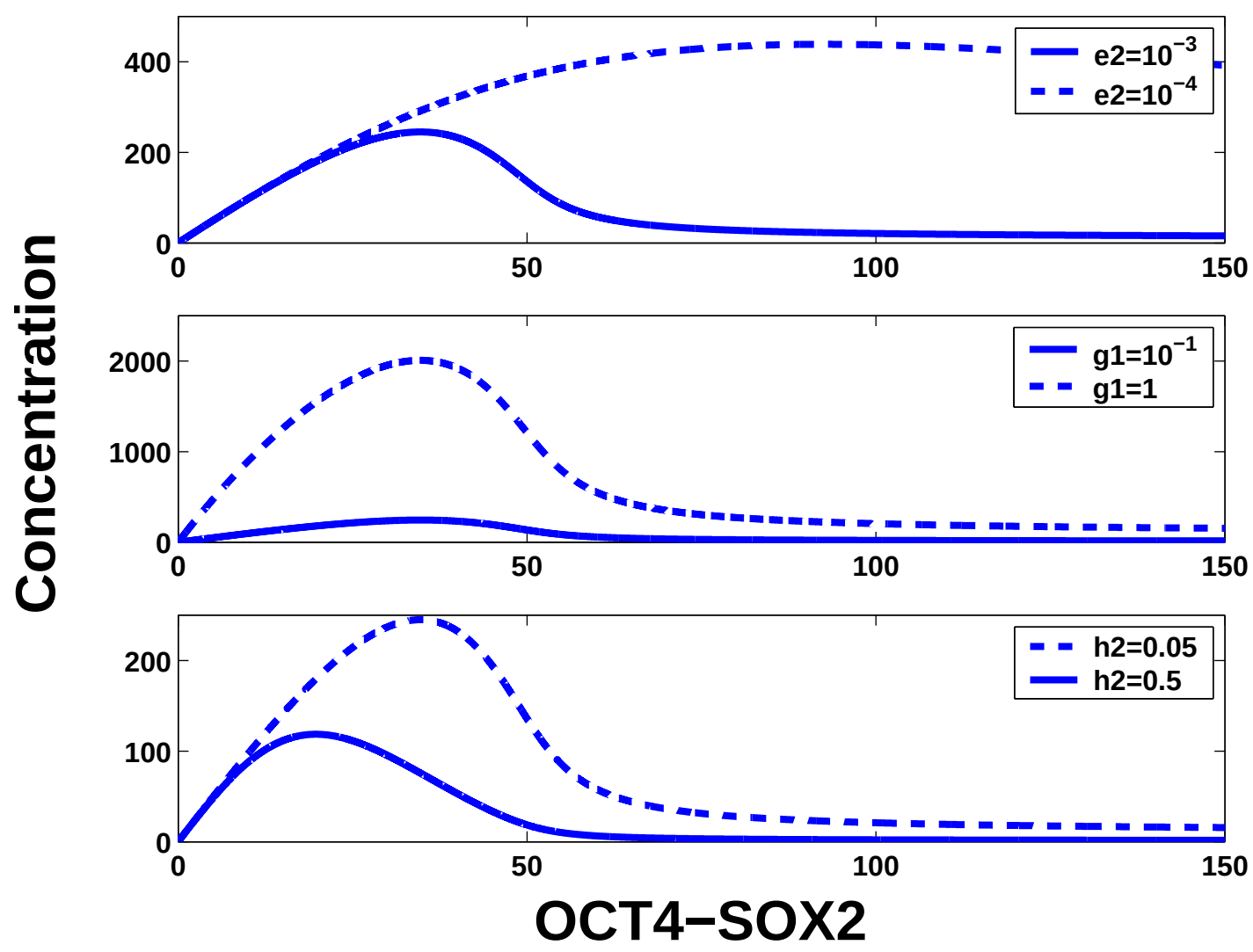

Figure S5: Target gene expression curves when varying parameters. Top panel: e_2 represents the strength of binding of OCT4–SOX2 and NANOG onto the NANOG gene, and therefore it represents the amount of autoregulation due to positive feedback. As we decrease the feedback, NANOG expression reduces, and hence the expression of the differentiation target genes increases. Middle panel: g_1 determines how strongly OCT4–SOX2 binds to the target gene. Hence for lower values of g_1, the target gene expression decreases. Lower Panel: h_2 represents the repression due to the OCT4–SOX2, NANOG complex binding to the target gene gene. Reducing the affinity of binding of the complex leads to a release of the expression of the differentiation target genes.

References

[1] Buchler NE, Gerland U, Hwa T (2003) On schemes of combinatorial transcription logic. Proc. Natl. Acad. Sci. USA **100**, 51365141.

[2] Chickarmane V, C. Peterson, Sauro HM (2006) Combinatorial Complexity of Transcription Factors leads to Interesting Circuit Design. Submitted.
